# Process Monitoring of Moisture Content and Mass Transfer Rate in a Fluidised Bed with a Low Cost Inline MEMS NIR Sensor

**DOI:** 10.1007/s11095-020-02787-y

**Published:** 2020-04-21

**Authors:** Claudio R. Avila, Joan Ferré, Rodrigo Rocha de Oliveira, Anna de Juan, Wayne E. Sinclair, Faiz M. Mahdi, Ali Hassanpour, Timothy N. Hunter, Richard A. Bourne, Frans L. Muller

**Affiliations:** 1 School of Chemical and Process Engineering,, University of Leeds, Leeds, LS2 9JT UK; 2 Department of Analytical Chemistry and Organic Chemistry, Universitat Rovira i Virgili,, 43007 Tarragona, Spain; 3 Department of Chemical Engineering and Analytical Chemistry, Universitat de Barcelona,, 08028 Barcelona, Spain; 4 Analytical Sciences,, GlaxoSmithKline,, Stevenage, UK

**Keywords:** fluidised bed drying, mass transfer resistance, MEMS Fabry-Pérot interferometer sensor, near infrared spectroscopy, online process monitoring

## Abstract

**Purpose:**

The current trend for continuous drug product manufacturing requires new, affordable process analytical techniques (PAT) to ensure control of processing. This work evaluates whether property models based on spectral data from recent Fabry–Pérot Interferometer based NIR sensors can generate a high-resolution moisture signal suitable for process control.

**Methods:**

Spectral data and offline moisture content were recorded for 14 fluid bed dryer batches of pharmaceutical granules. A PLS moisture model was constructed resulting in a high resolution moisture signal, used to demonstrate (i) endpoint determination and (ii) evaluation of mass transfer performance.

**Results:**

The sensors appear robust with respect to vibration and ambient temperature changes, and the accuracy of water content predictions (±13 % ) is similar to those reported for high specification NIR sensors. Fusion of temperature and moisture content signal allowed monitoring of water transport rates in the fluidised bed and highlighted the importance water transport within the solid phase at low moisture levels. The NIR data was also successfully used with PCA-based MSPC models for endpoint detection.

**Conclusions:**

The spectral quality of the small form factor NIR sensor and its robustness is clearly sufficient for the construction and application of PLS models as well as PCA-based MSPC moisture models. The resulting high resolution moisture content signal was successfully used for endpoint detection and monitoring the mass transfer rate.

**Electronic supplementary material:**

The online version of this article (10.1007/s11095-020-02787-y) contains supplementary material, which is available to authorized users.

## Introduction

Near infrared spectroscopy (NIR) has been established as a key tool for process analysis technology (PAT) (1). For pharmaceutical applications, it has proven to be an effective technique for gathering relevant chemical data to build up process understanding (2,3), contributing to new process development (4), and for process monitoring and control during the drug manufacturing processes (5). Recent advances in instrumentation and chemometrics have been identified as the main pillars advancing NIR spectroscopy towards application in manufacturing, but cost and measurement robustness remain barriers to widespread implementation in the pharmaceutical industry.

The recently developed Micro-electro-mechanical system Fabry–Pérot Interferometers (MEMS FPI) for near infrared wavelengths are miniaturised tuneable optical filters formed by two facing reflectors separated by an air gap. The distance between the two reflectors is controlled by the voltage applied (6). Light with a wavelength of the gap size will interfere and pass the filter and be collected by a single-point detector positioned below. The range of distances the device can set will thus determine the range of wavelenghts the device can measure (7). MEMS-FPI based devices with different reflector gap widths target specific regions of the NIR spectral ranges (e.g 1.7*μm*, 2*μm*). For a specific process application, the appropriate spectral range can be matched with the sensor range (8–10).

The resolution of the MEMS FPI sensors is lower than in Fourier Transform NIR spectrometers or traditional diffraction gratings spectrometers, but the compact form factor and cost-effective pricing enable new applications that are not possible with aforementioned traditional spectral sensing technologies. To measure spectra, the voltage is changed gradually and the detector signal is recorded. Spectral data can be recorded up to speeds of 1000 spectral points per second, providing the user a high rate of data at a few spectral positions, or a full spectrum over the range available at a lower rate.

MEMS-FPI are made from a single wafer without assembly steps, creating a single solid structure with no wearing parts which makes the devices position and vibration insensitive; staying very stable over time. Developed low-cost MEMS-FPI and detector modules have been successfully miniaturised, with systems weighing as little as 60 g (11), making them suitable for widespread application in process sensors in the manufacturing industry. This would be a major step forward to continuous, inline composition measurement.

For the pharmaceutical industry, accessing reliable spectral information at a low cost could bring immediate benefits. For instance, complementary compositional and physical information could be obtained for several drug manufacturing stages before attempting to replace conventional quality control or research analytical systems. A specific example is the monitoring of moisture content, a control parameter used in several stages of the solid-dose form production process (5), normally required for milling and blending (12,13), granulation (14,15), tablet coating (16), and dying, particularly fluidised bed granule drying, which has been extensively studied (17–20).

In examples reported, real-time moisture determination using NIR spectroscopy relies on correlating online NIR spectra to offline analytical moisture measurements (typically Karl Fisher titrations or loss on drying (LOD) (14,21)). Partial Least Squares regression (PLS) is the algorithm of choice to model the measured moisture content (vector **y**, *n* data points) as a linear combination of *n* measured spectra (matrix **X**, one spectra on each row^1^):

1 $$\mathbf{y}=\mathbf{X}\ \mathbf{b}+{b}_o+{\mathbf{e}}_y$$

where **b** the vector with relative contributions of each spectral point, *b*_*o*_ is a constant (22) and **e**_*y*_ is the vector of errors. With **b** and *b*_0_ obtained from a calibration data (**y**, **X**), a real-time moisture content signal can be inferred from a new NIR spectrum by application of Eq. 1 (23).

The second common application of NIR data is for drying endpoint detection. Here, Multivariate Statistical Process Control (MSPC) models based on Principal Component Analysis (PCA) are built from sets of spectra of different Normal Operating Condition (NOC) batches that represent the process end-point well. In-line spectra from new batches are tested in real time to determine whether they behave, or not, as the NOC spectra used to build the model (24,25).

To build MSPC models for process end-point detection, a data set formed by NIR spectra of on-specification batches where the end-point has been reached are used in a matrix X containing of *n* end-point spectra. A PCA model is built in order to set the statistical boundaries of the experimental domain (space) of end-point NIR spectra (2,3):

2 $$\mathbf{X}=\mathbf{T}\ \mathbf{P}+\mathbf{E}$$

where **P** is the loadings matrix (nr of principal components, nr of wavelengths which are the link between scores and original NIR spectra) and **T** is the scores matrix of all end-point spectra (nr of spectra, nr of principle components). **T** spans the valid experimental domain for on-specification measurements in the space of principal components. The matrix **E** describes the residual errors of the PCA model. For any new (pre-processed) spectrum **x**_new_ acquired in the current on-line monitored batch, the difference between the spectrum **x**_new_ and its description by the PCA model **x**_new_**P**^T^**P** is:

3 $${\mathbf{e}}_{\mathrm{new}}={\mathbf{x}}_{\mathrm{new}}\ \left(\mathbf{I}-{\mathbf{P}}^{\mathrm{T}}\mathbf{P}\ \right)$$

***e***_*new*_ is a row vector containing the residual error for each wavelength. *Q*_*stat*_ control charts are developed on this basis of the sum of squares of this error:

4 $${Q}_{stat}={\mathbf{e}}_{\mathbf{new}}\ {\mathbf{e}}_{\mathbf{new}}^{\mathbf{T}}$$

When *Q*_*stat*_ falls below a minimum error, the chart control limit, the new spectra resembles the typical endpoint spectrum shape as defined by the NOC batches.

Acquiring online spectra from multiphase processing equipment (air-solid) (26) such as fluidised bed dryers, wet granulators and excipient blenders, presents several challenges such as noise and probe window fouling. For instance, within a fluidised bed, drying granules will flow past the NIR probe window, and particles will interact with the probe’s NIR light at a wide range of distances and orientations. The significant changes in material density and air gaps with variable distance between the solids and the field of view of the probe results in an intermittent signal (27). These interactions lead to significant levels of noise that distort the coefficients in Eqs. 1 and 2–4 resulting in large residual errors.

Under these conditions, it is essential to reduce the noise introduced during spectral measurement by suitable pre-processing of the NIR spectra (4,28), for instance by averaging a number of them.

This study aims to evaluate the accuracy, robustness and reliability of the spectral response obtained from the MEMS-FPI NIR sensor by monitoring the moisture content during the drying of pharmaceutical granules in a pilot-scale fluidised bed dryer. PLS models resulting from a validation data set transform online spectral measurements to predictions of the moisture content and MSPC tools use spectra for detecting the process end-point. In the final part, we convert the NIR derived moisture content signal to the water mass transfer rate and demonstrate how this can be applied so as to provide an insight in to the underlying processing phenomena.

## Materials and Methods

### Granulation and Drying

Pharmaceutical granules were produced using a standard recipe supplied by GlaxoSmithKline (GSK, United Kingdom) employing Mannitol (64 wt%, solid wt% are with respect to the dry granule weight), Microcrystalline Cellulose Avicel PH-101 (29 wt%), Hypromellose 2910 (5 wt%, binder), AC-DI-SOL (1.5 wt% disintegrant) and colloidal SiO_2_ (0.5 wt%). A top driven high shear mixer wet-granulator model MiPro from ProCepT (Belgium) was used to prepare fourteen 1 kg batches of granules in a 2 l vessel with a three-bladed impeller rotating at 800 RPM. During granulation, Water (0.5 L/kg_solid_) was added at a constant feeding rate of 5 mL/min. Due to intense mixing the temperature rises from room temperature to approximately 45°C. On completion of the granulation, granules are transferred into a container and sealed up before cooling down and storing in a fridge at 5°C to minimise changes in moisture content before drying. Batch-to-batch repeatability in terms of moisture content and particle size was achieved by using the granulator torque profile as an online indicator, in parallel to a correlation between the water addition with the particle size (using an image analysis method) obtained from a control batch. This methodology mirrors a granulation procedure previously reported (29) and further information related to the granulation steps is provided in the Supplementary Material.

**Fig. 1:**
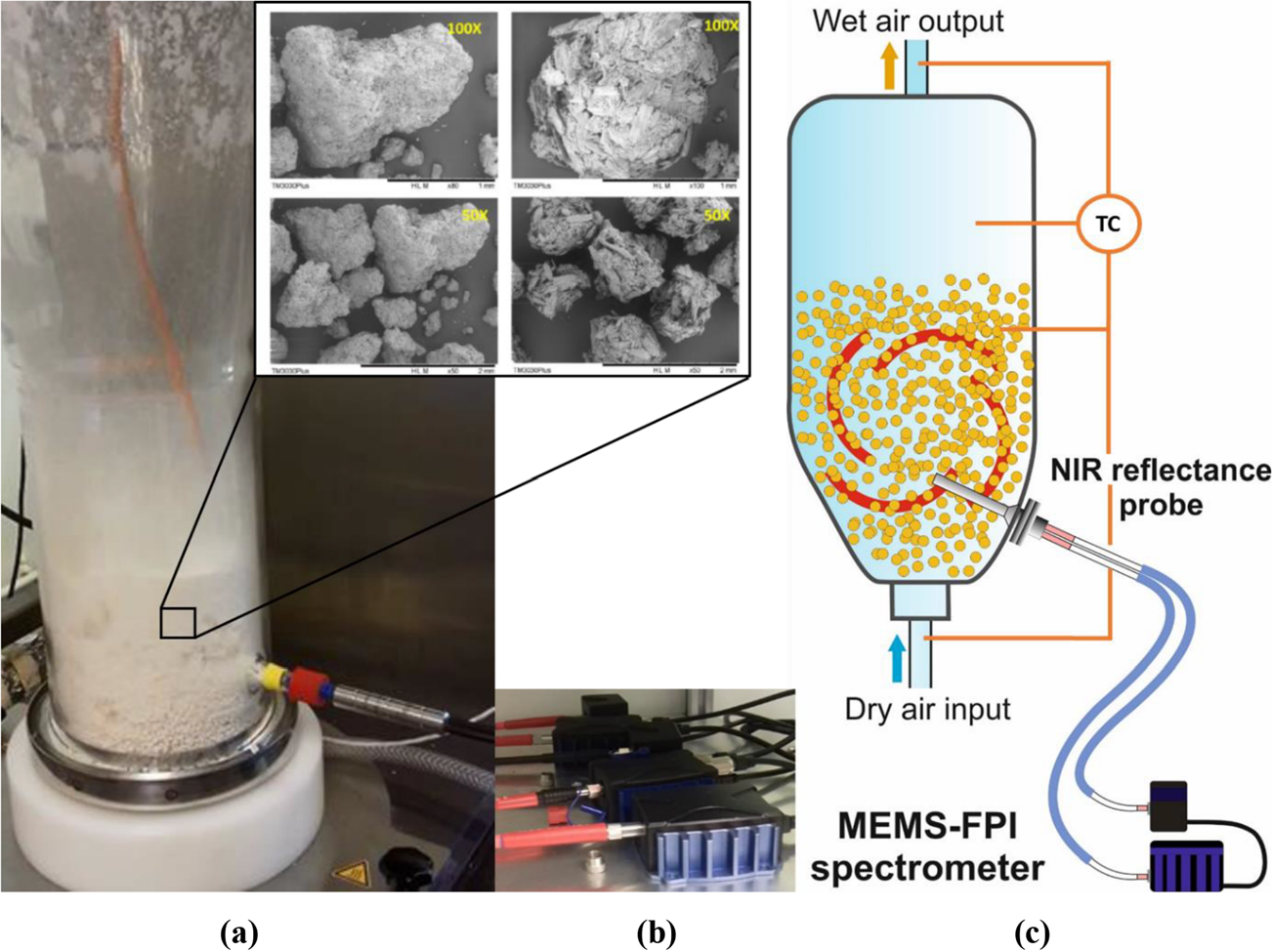
(**a**) The fluidised bed dryer with NIR immersion probe attached (**Top**) SEM pictures of granules after the drying process, (**b**) the spectral sensor and light source connected to light guides. (**c**) Schematic view of the system including the position of the MEMS-FPI sensor used, NIR probe location, and temperature (TC) measuring points (right).

Drying was performed in a 0.129 m ID, 4 l fluidised bed model 4 M8-Trix Formatrix from ProCepT (Belgium). A picture of the equipment used is shown in Fig. 1, and a summary with the experimental drying conditions of each batch is presented in Table I. For each batch, 0.5 kg of wet granules (1 l equivalent) were manually transferred into the fluidised bed, and continuously dried by passing a constant flow of air at 25 ± 3°C to fluidise the granule bed. The air flow was set to 850 L/min for batches 1 to 10 and to 600 L/min for batches 11 to 14. Visual observation indicates that at both the air flowrates the fluidised bed operates in the bubbling regime, though the NIR signal readings were smoother at the lower gas flowrate.

**Table I:** Summary of Experimental Conditions for Fluidised Bed Drying of Granules Spectra where Recorded at 1 Scan per Second

Sample name	PLS Model	Moisture drying range (from/to)	Flow rate L/min	Recording time (min)	time to reach 5% (min)^**^
Batch 1	Calibration	35.03% to 1.99%	850	137.3	58
Batch 2	Calibration	34.11% to 1.77%	850	117.1	46
Batch 3	Calibration	34.45% to 1.86%	850	123.3	54
Batch 4	Calibration	34.24% to 1.85%	850	108.8	42
Batch 5	Calibration	37.60% to 1.86%	850	112.5	43
Batch 6	Calibration	33.94% to 1.51%	850	114.5	39
Batch 7	Validation	33.55% to 1.32%	850	78.0	38
Batch 8	Validation	33.89% to 1.46%	850	85.1	39
Batch 9	Validation	33.83% to 1.62%	850	86.8	41
Batch 10^*^	Validation	33.92% to 1.63%	850	79.5	40
Batch 11	Validation	34.09% to 1.88%	600	88.0	49
Batch 12	Validation	34.30% to 2.55%	600	117.7	78
Batch 13	Validation	33.88% to 2.18%	600	100.1	64
Batch 14^*^	Validation	33.42% to 1.86%	600	116.3	66

*After each manual sampling fluidisation was switched off for 10 s

**Estimated time since the fluidisation started up to reaching 5% of moisture content

Every 6–7 min a sample of approximately 5 g was retrieved from the fluidised bed using a vacuum suction for offline moisture analysis. Sampling required the NIR probe to be disconnected from the vessel for approximately 10 s (to make sampling port available). For batches 10 and 14 the fluidisation air was off for 15 s after each manual sampling, allowing collection of NIR spectra with reduced fluctuations in particle density and orientation.

### Analytical Characterisation

Reference moisture content (loss on drying, LOD) was measured using a thermogravimetric moisture analyser model MB120 from Ohaus (Germany), operating at a constant temperature of 105°C. Granule samples obtained from the fluidised bed were directly deposited in the MB120 sample chamber and dried in a 5 to 10 min period (depending on the moisture content).

The LOD moisture fraction (*f*_*w*_) reported is defined by the weight of water (*m*_*w*_) and dry weight of the solid (*m*_*S*_) as follows:

5 $${f}_w=\frac{m_w}{m_w+{m}_s}$$

In and outlet temperatures were recorded simultaneously to the NIR spectral measurements using K type immersion thermocouples from Omega (United Kingdom) connected to a TC-08 AD converter from Pico technologies (USA). The gas flowrate is measured by the ProCepT control system.

### MEMS-FPI NIR Sensor and Data Acquisition

**Fig. 2:**
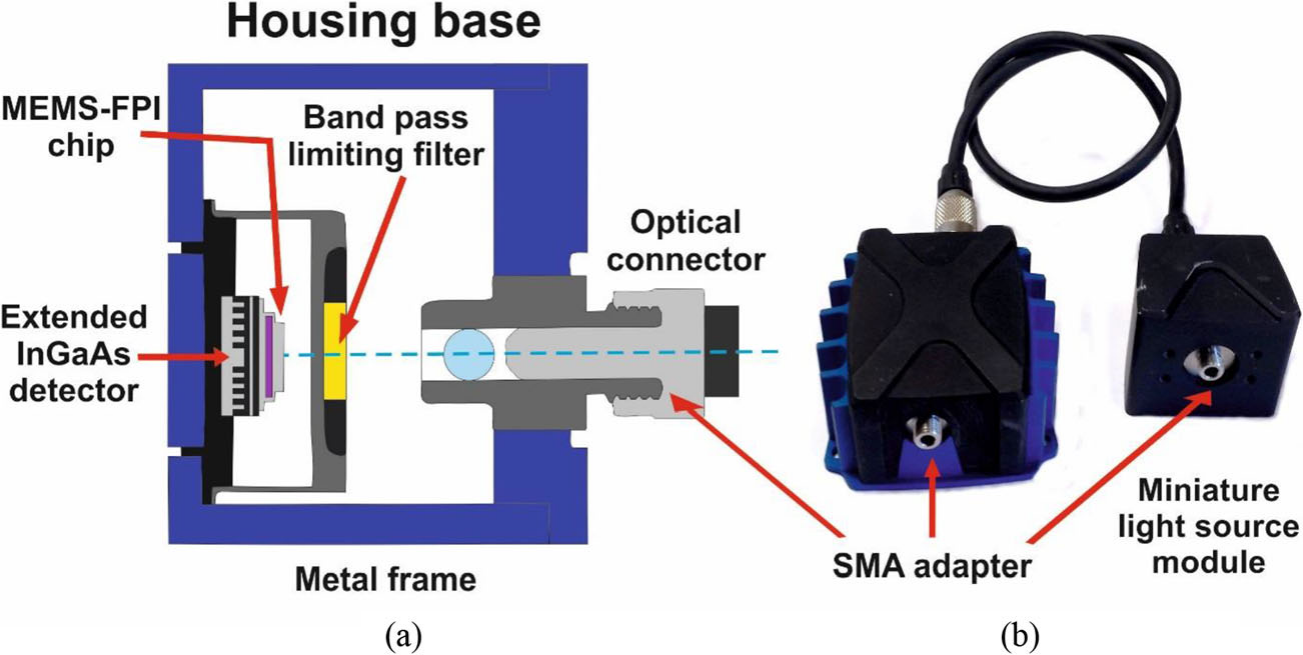
The tuneable MEMS Fabry–Pérot Interferometer: (**a**) schematic of the internals showing the MEMS FPI chip mounted on top of the InGaAs NIR detector and (**b**) and a photo of the miniaturised NIR sensor with the light source. The footprint of the assembled sensor chassis is approximately 58 mm length by 57 mm width by 27 mm high, with a weight of 125 g.

A sensor from Spectral Engines (Finland) model N-Series 2.2, operating from 1750 nm to 2150 nm wavelength range was used for the acquisition of NIR spectra (ca. 4650–5714 cm^−1^). It has a tuneable MEMS Fabry–Pérot Interferometer acting as the spectral element and a single element extended InGaAs detector (Fig. 2; additional information about the scanning principle of the device can be found in the Supplementary Material). The sensor has an integrated light source model LS-PRO that utilises a miniature tungsten vacuum lamp as the illumination source. The energy output of the lamp was set to 50% of the maximum level. For all drying batches, the integration time of the sensor was set to 0.1 ms and the wavelength step to 1 nm (10 ms to set a step). This results in 401 wavelength points which including data transfer time results in an acquisition time of approximately 1 full NIR spectrum per second (single scan, no averaging). Control and communication of the NIR sensor, data logging of the NIR spectra and recording of temperature readings, were performed using a bespoke application developed in LabVIEW 2015 by the University of Leeds (ChemiView version 3.4 (30)).

### NIR Reflectance Probe and Calibration

A 6 mm diameter immersion NIR diffuse reflectance probe model OFS-6S-100HO/080704/1 from Solvias (Switzerland) was inserted horizontally with the probe’s tip located 45 mm above the bottom and in the centre of the bed (Fig. 1). The probe has a stainless steel body with a sapphire window and contains two fibre optic cables, one connecting to the light source, the other to the NIR sensor using standard SMA adaptors. A bright reference spectrum was obtained before starting each batch. A calibration block was used to position the tip of the NIR probe in a 90-degree angle relative to a diffuse reflectance standard (model Spectralon USRS-99-010 from Labsphere, USA).

### Chemometric Modelling

Chemometric modelling and validation were carried out with in-house routines programmed in Matlab R2017a (Mathworks, USA) and with PLS_Toolbox 8.5.1 (Eigenvector Research, USA) running under Matlab.

#### Spectral Preprocessing

Intensive preprocessing was required to remove the spectral artefacts generated as result of inhomogeneity in the fluidised bed system. First, 10 intensity raw spectra were averaged into a single raw spectrum. The resulting signal was transformed into absorbance using the bright and dark reference spectra. As artefacts are still present in the data, a moving average filter was applied using the current and 74 prior spectra. Finally, spectra were mean-centred before being submitted to the PLS algorithm. The Standard Normal Variate method was evaluated, but in this case did not improve over the pre-processing method described.

#### PLS Regression for Moisture Prediction

The PLS regression model was built relating the NIR pre-processed spectra (**X**) to the mean-centred logarithm of LOD moisture content of samples collected during the drying of the 6 initial batches ($y={\log}_{10}{f}_w-\overline{\log_{10}{f}_w}$**;** Batches 1 to 6, see Table I). The log_10_ transformation was used to minimise the relative error; the error is large at high values of *f*_*w*_ due to sticking etc. and reduces as the system dries. This improved the predictions over the wide range of moisture content (1.51% to 37.60%). The PLS regression model was calculated using the NIPALS algorithm (31). Finally, PLS outputs were back-transformed to provide moisture values in the original units. Note that when applying Eq. (1) to calculate the log moisture content *y* for a new spectrum, the error in Eq. (1) represents a relative error in the *f*_*w*_ domain; i.e. the expected moisture content ${f}_w(X)={e}^{y+\overline{\log_{10}{f}_w}}\times {e}^{\pm {e}_y}\approx {\left.{f}_w(X)\right|}_{est}\times \left(1\pm {e}_y\right)$.

#### MSPC Charts for Process End-Point Detection

PCA-based MSPC model charts were built using NIR spectra corresponding to an offline determined moisture content below 2% chosen as the process end-point criterion. The MSPC model was built using NIR spectra from batches 2, 3, 4, 6, 7, 9 and 10, that each reached the desired process end-point i.e. moisture content below 2%, using the same pre-processing as for the PLS model. All the spectra were gathered in matrix ***X*** (nr. of spectra, each with 400 wavelengths). After this step, a standard normal variate (SNV) normalisation was applied to remove any unwanted baseline spectral variation followed by mean-centring of the ***X*** matrix before building the PCA-based MSPC model. The number of components in the PCA model was estimated by cross-validation. Finally, a *Q*-statistic control chart (*Q*_*stat*_) was built from the resulting MSPC model and control limits at 95 and 99% confidence interval were estimated according to Jackson and Mudholkar equation (32). For external validation, *Q*_*stat*_ control charts were obtained for batches 1, 5, 8, 11, 12, 13 and 14 (not used in the PCA model building step). When the shape of a new spectrum is similar to the end-point spectra used to build the PCA model, the residuals are small and the related *Q*_*stat*_ value appears below the chart control limit. Conversely, when a spectrum is far from the end-point, the spectral shape is clearly different and the resulting *Q*_*stat*_ value appears well above the chart control limit. The point in time where the *Q*_*stat*_ value goes below the control chart limit at a 95% confidence interval for 10 consecutive observations was used as criterion to indicate the process end-point. Batches that do not reach the end-point should consistently show *Q*_*stat*_ values above the chart control limit. It is important to remind that the end-point detection by MSPC uses the sole information provided by the NIR spectra and does not require any reference moisture content.

## Results and Discussion

The first aim of the work is to assess the robustness of the new reduced cost and small form factor MEMS FPI NIR sensor over a 9 month period during which 14 batches of placebo granules were manufactured and dried in a fluidised bed (Table I). The objective was to repeat essentially identical batches in order to evaluate the robustness and consistency of the sensor, and the predictions based on models derived from the NIR spectra. The system was however subject to changes uncontrolled variables: such as batch to batch variations in granulate (e.g. size, moisture content, storage time) and the ambient and air inlet temperature and humidity (experiments started with bx1 in August to bx 14 in mid December). To probe the sensitivity of the PLS moisture predictions to a change in flowrate we reduced the air flowrate from 850 to 600 L/min in the last 4 batches.

Overall, the MEMS-FPI sensor performance showed a very satisfactory stability and reproducibility. The spectral signal remained stable and repeatable under all ambient conditions. The bright reference intensity levels and spectra shape measured at the beginning of each experiment remained similar. The device proved to be free of interferences generated by mechanical vibrations; e.g. spectral readings did not alter when the device was installed directly next to the fluidised bed vessel. Ambient temperature variation did lead to minor variations. However, using a second spectrometer it was demonstrated that this was in fact due to effects of temperature on the transmission through the fibre optic cables, rather than on the light source or the MEMS-FPI sensor.

**Fig. 3:**
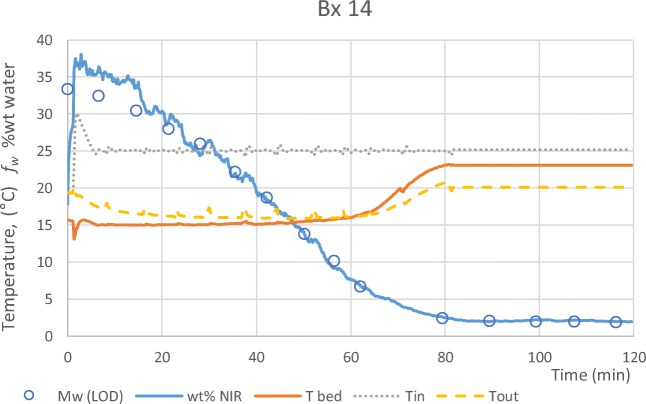
A typical data set for a drying experiment (Bx 14). Granules are charged cold and fluidised by a gas stream of 600 L/min at ~25 ° *C*. As water evaporates, heat is removed from the gas stream resulting in a ~10 ° *C* temperature difference between the bed and the fluidising gas. Moisture content is monitored by NIR using the PLS model (Eq. 1), and measured offline by LOD. When the granule water content is reduced by 85% the evaporation rate drops and the bed temperature rises. Equilibrium between the inlet gas (ambient RH) and the granules (~1.5 wt% water) is reached after ~90 min.

In the fluidised bed process granules are dried using ambient air of uncontrolled humidity. As a result the drying rate varies from batch to batch, with the final drying end-point determined by relative humidity conditions found on the day when a batch is dried. Table I summarises the observations for all batches. A typical data set for a drying experiment is shown in Fig. 3: Granules are charged cold and fluidised by a gas stream of 600 L/min at ~25 ° *C*. As water evaporates, heat is removed from the gas stream resulting in a ~10 ° *C* temperature difference between the bed and the fluidising gas. The moisture content measurement is based on a PLS model (Eq. 1) based on 6 reference batches and validated using the NIR spectra from a further 8 validation batches. Moisture content is monitored continuously by NIR using the PLS model, and measured once every ~10 min by sampling and performing offline analysis by LOD. When the granule water content is reduced by ~85% the evaporation rate drops and the bed temperature rises. Equilibrium between the inlet gas (ambient RH) and the granules (*f*_*w*_≈1.5–2 wt%) is reached after ~90 min at 600 L/min.

To test the robustness of the NIR measurement and the associated PLS model, drying was performed using two different flow rates (600 and 850 L/min) at constant air inlet temperature (25 ± 3°C). As expected, the flowrate strongly affects the drying rate: thus, the drying time reduces to ~60 min when the flowrate is set at 850 L/min. Even though the PLS model is based on data from runs 1 to 6, all operated at 850 L/min, it still correctly predicts the moisture content of runs at 600 L/min without further chemometric analysis, since only the drying rate, but not the sample composition, is changed. Thus, the fitted parameters for Eq. (1) successfully correlate *f*_*w*_ to the NIR spectra for the reference batches, and the same parameters allow prediction of moisture content for all validation batches irrespective of the flowrate. This demonstrates (i) the robustness of the data strategy applied (e.g. data treatment, data treatment prior to PLS analysis) and (ii) the excellent stability and robustness of the MEMS-FPI NIR sensor.

When drying from ~33 to 10 wt% moisture contents it was observed that the granules shrank due to the loss of 26% of their original mass. At this stage no significant fines were observed. At lower moisture levels (5–1%), granules appear dried at the surface. Granule size continue to reduce, but now by attrition which generated a significant quantity of fines that were observed to build up on the fluidised bed filters, resulting in increasing pressure drop across the outlet filters. The size reduction, coupled to the reduction in moisture content did appear to increase the intensity of the reflected signal as the drying process progressed, but this did not seem to have a significant impact on the moisture content predictions.

**Fig. 4:**
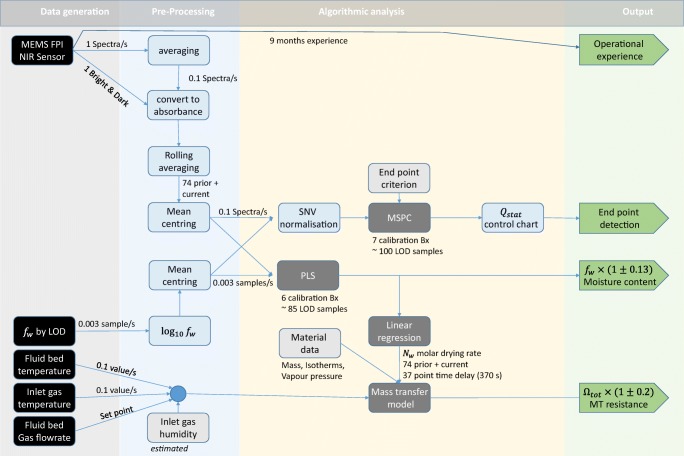
An overview of the sensor data analysis strategy used to demonstrate the versatility of the new MEMS FPI NIR sensor. Data generated by sensors is pre-processed and delivered to algorithms to construct models from calibration data. With these models are in place, new NIR spectra recorded can be interpreted immediately.

A data analysis strategy was designed (Fig. 4) to demonstrate that the low cost NIR sensors delivers high quality data robustly during fluid bed drying on placebo pharmaceutical granules; a typical processing scenario with a very low signal to noise ratio. We tested three applications: Moisture monitoring, end-point detection and process analysis (mass transfer monitoring) that will be discussed in more detail below

### NIR Signal Acquisition during Fluidised Bed Drying

**Fig. 5:**
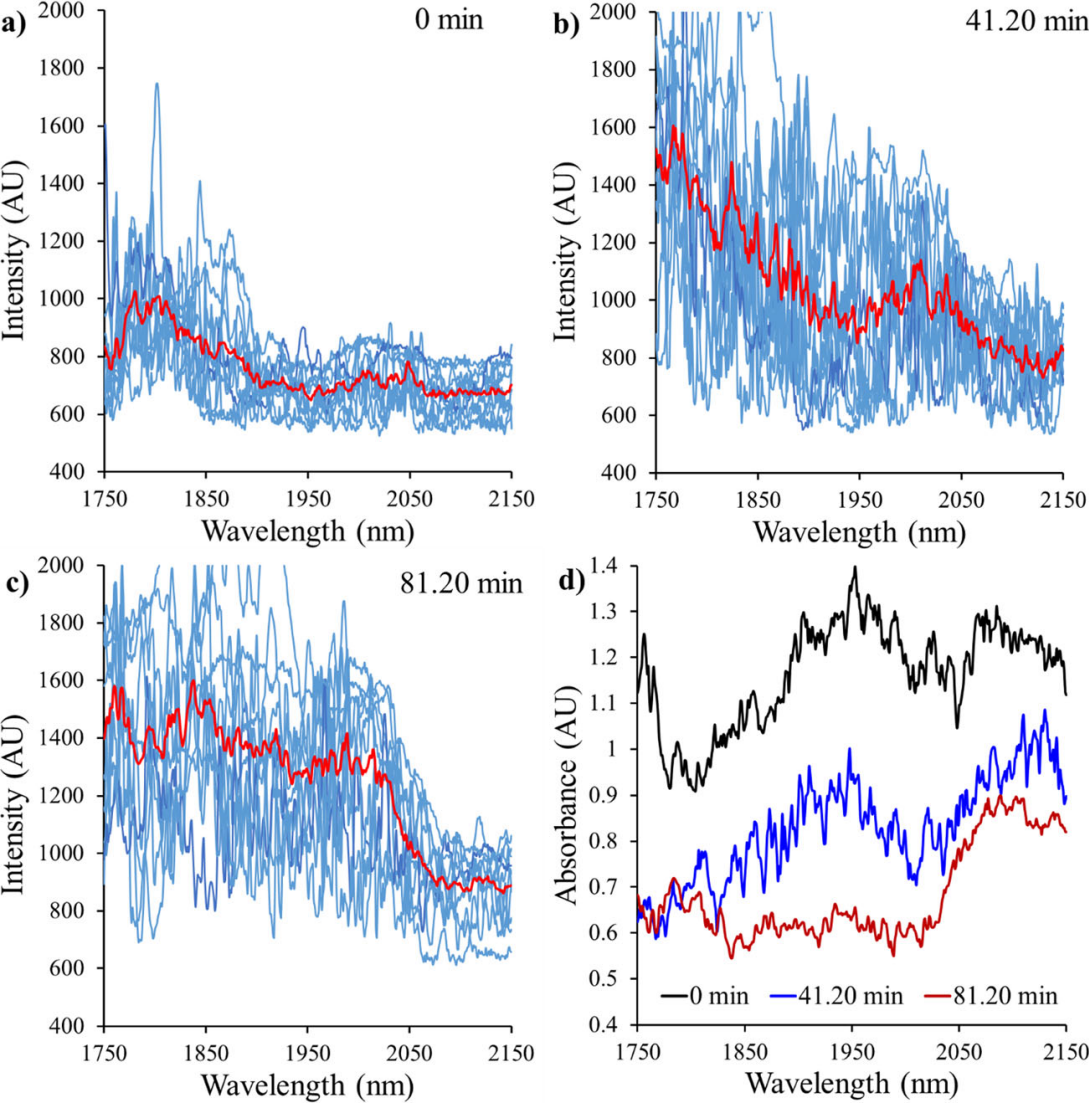
Construction of absorbance signal from the average (red line) of 10 consecutive single scans (blue thin background lines) for three time periods of the drying process: (**a**) t = 0; (**b**) t = 41.2 min; and (**c**) t = 81.2 min, (**d**) Absorbance spectra obtained from the resulting average intensity. Dark signal level of the detector was approximately 500 intensity units.

The presence of large fluctuations in individual NIR scans when sensing *in situ* from a fluidised bed dryer has been previously reported and necessitates intensive data pre-processing steps and/or modification of the way a spectrum is measured (e.g. scoop devices to hold the sample in place while measuring) (28). The contact between the fluidised material and the tip of the NIR probe is variable and characterised by air gaps appearing in front of the sensing area at random intervals. This produces abrupt changes in the spectra collected from scan to scan. Figure 5a, b, c shows 10 consecutive single spectral readings and their corresponding average (darkened line) for three different time periods of the drying process (a, t = 0 min; b, t = 41.2 min; and c, t = 81.2 min). In this work 10 single spectral scans were averaged to give a single NIR spectrum every 10 s. This procedure is exemplified in Fig. 5, where the average of the 10 single scans was converted to the absorbance spectra. The resulting absorbance signal still shows a relatively high level of noise, which is further reduced with a moving averaging filter that averages the data of the currently aquired spectrum with the previous 74. The large filter window (750 s) provides a smoother variation of the spectral observations over time and only slightly reduces the response time of the moisture content predictions.

**Fig. 6:**
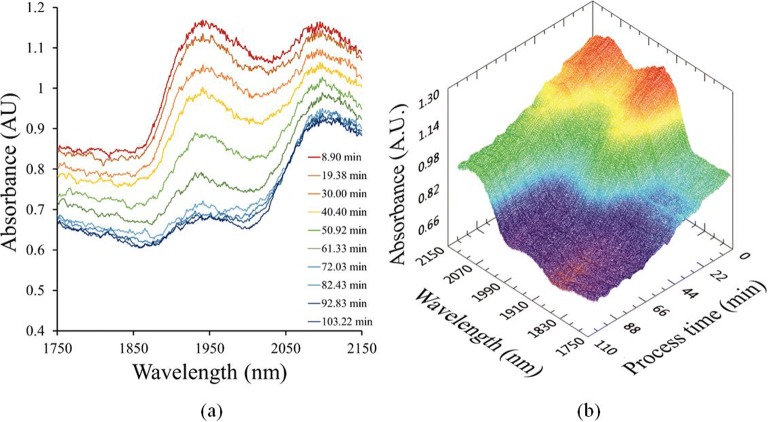
Change in water content observed from variations in the absorbance spectra (**a**): for 10 specific periods of the drying process (obtained after applying the pre-processing steps). (**b**): NIR profile evolution observed for the complete drying process.

After the pre-processing steps, the drying evolution can be clearly observed in the spectra (Fig. 6): the absorbance of the -OH absorption band (1940 nm), associated with the presence of water in the material, strongly reduces as drying progresses.

Fouling is another issue observed when acquiring *in situ* NIR spectra in a fluidised bed system. This problem often occurred at the beginning of the drying process, since granules with a moisture content above 20 wt% are very wet, and have a strong tendency to stick to the probe’s window. This causes more reflection, and thus higher intensity NIR spectra, which shifts the signal to greater values compared to normal operation with a clean window. Fortunately, continuous collision of granules on the probe window causes a degree of self-cleaning. Hence, if granules stick for periods significantly smaller then 750 s, the pre-processing steps will reduces the impact of fouling on moisture content measurements.

### Moisture Content Prediction with the NIR Sensor

A PLS model was developed by relating the pre-processed NIR spectra from the initial six batches (Table I), to the offline measured LOD moisture content. For the six calibration batches (Bx1 to 6), different, narrow spectral regions including the spectral range corresponding to the strong moisture band (1900–2000 nm) were tested separately but the smallest overall moisture prediction error was found using the full spectral range available from the sensor (1750–2150 nm).

**Fig. 7:**
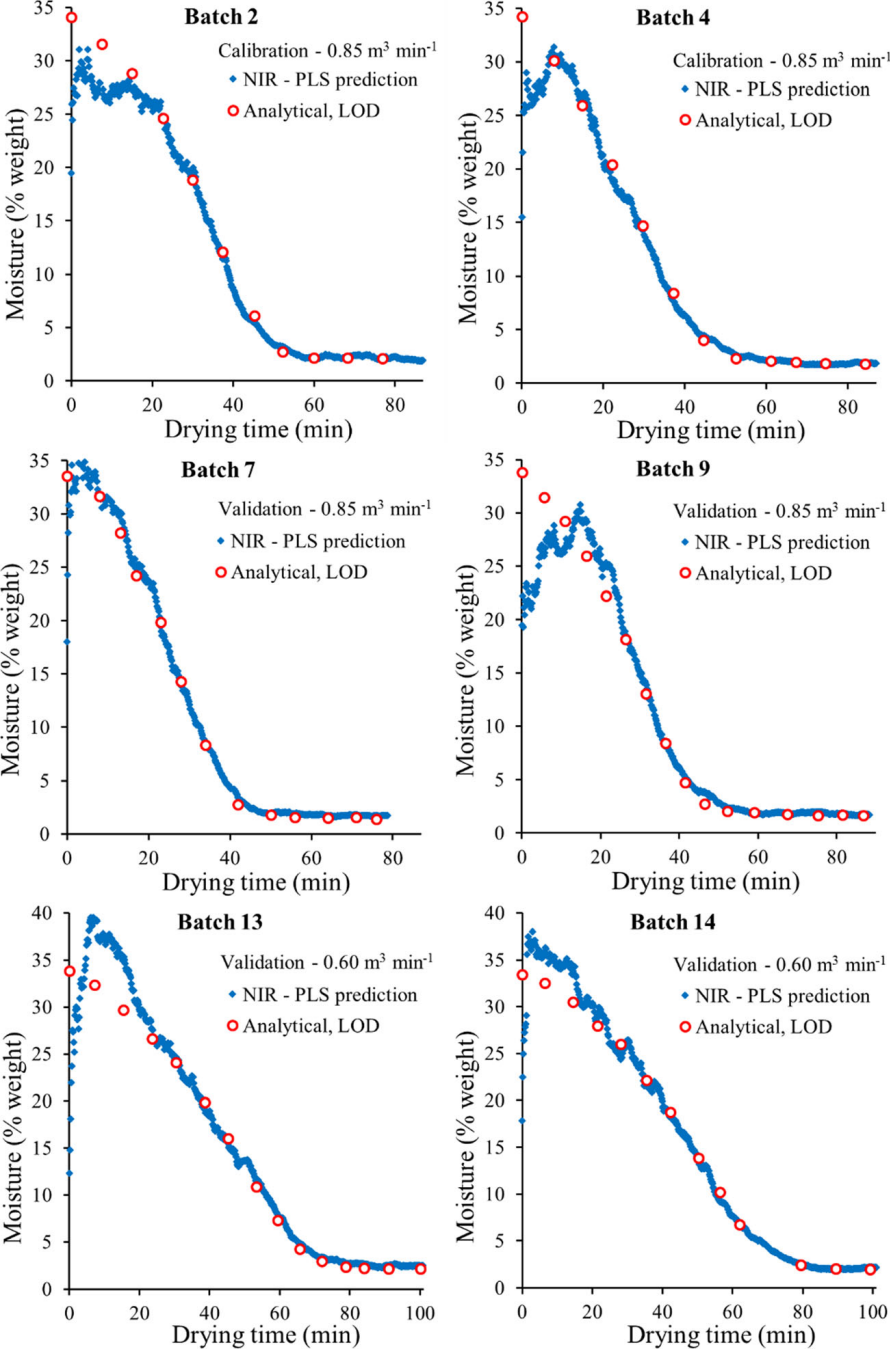
Analytical moisture content determined using LOD (discrete circles) compared to the prediction profiles obtained from the PLS regression model using online NIR spectra (semi continuous line), including two calibration batches (batches 2 and 4 using 850 L/min), and four validation batches using two flow rates (batches 7 and 9 using 850 L/min; batches 13 and 14 using 600 L/min).

Figure 7 shows the results obtained for six selected batches comparing the LOD analytical moisture content (circles) with the predicted moisture profiles obtained from the online NIR spectra and the PLS regression model (continuous line). The figure shows (i) two calibration batches used for developing the PLS model (batches 2 and 4, using 850 L/min), (ii) two validation batches with the same flow rate (batches 7 and 9, using 850 L/min), and (iii) two validation batches using a slower flow rate (batches 13 and 14 using 600 L/min). Similar plots showing the results obtained for all fourteen batches can be found in the Supplementary Material. Generally, the moisture content resulting from the NIR spectra provides a good estimate of the data measured offline by LOD when the moisture content *f*_*w*_ < 20*wt*%.

**Fig. 8:**
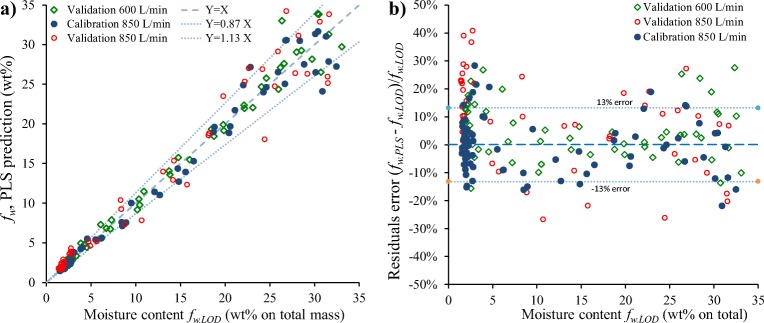
Comparison of the moisture content *f*_*w*. *PLS*_, as measured by the NIR sensor with the PLS statistical model, and the analytical LOD data *f*_*w*. *LOD*_. (**a**) direct comparison and (**b**) relative residuals of the prediction, {(*f*_*w*. *PLS*_ − *f*_*w*. *LOD*_)/*f*_*w*. *LOD*_}.

A direct comparison of the moisture content measured using LOD with the predicted results from online NIR spectra is given in Fig. 8. As the PLS model fitted log_10_*f*_*w*_, we expect the relative error in the prediction to be constant for different levels of water content.

The average residual for all *N* LOD measurements (~200 *LOD values*) may be defined as:

6 $${e}_{rel}=\sqrt{\frac{1}{N}\sum \limits_{i=1\ to\ N}{\left(\frac{f_{w. NIR}-{f}_{w. LOD}}{f_{w. LOD}}\right)}_i^2}=13\%$$

From Fig. 8 it is clear that the error in the NIR based water content is independent of the absolute extent of the moisture content. The error for the calibration batches is ± 10%. However, all models over predict the LOD concentration below 5 wt%: 1% higher for the calibration batch, 18% for the 850 L/min and 8% for the 600 L/min validation batches. The high deviation at low water levels for the 850 L/min batches may be due to an uncontrolled parameter such as the humidity.

Comparing results to previous studies is difficult as the water concentration ranges used to build principal component models vary significantly. The pre-processing strategy was similar in all cited cases, averaging a large number of scans (from 32 to 300 vs 750 in this study) to compensate for the spectral noise. Peinado *et al*. (23) reported data for water contents *f*_*w*_ between 0.6 wt% to 2.8 wt% with a relative error *ε*_*rel*_ ≈ 15% using an ABB Fourier Transform Process Analyser Near Infrared spectrometer, with thermo electrically cooled InGaAs detectors (ABB-FTPA2000260). Fonteyne *et al*. (19) reported data for *f*_*w*_ in the range of 3.5 to 7 wt% with a relative error *ε*_*rel*_ = 5% using a Matrix™ –F Duplex, Bruker Optics Ltd., FT-NIR spectrometer (32 single scans averaged; using 1000–2220 nm for analysis). The residuals obtained using a commercial dispersive spectrometer varied ±4 wt% over 4-20 wt% water content (32 single scans averaged; using 1100–2500 nm for analysis) (28). The results obtained for the calibration and the validation batches with the novel MEMS-FPI sensor (*ε*_*rel*_=13%) were similar to those reported with conventional spectrometers. We did however observe significantly larger relative errors below 5 wt% water in the validation batches. The absolute errors remained low (~0.4 wt%). Based on the operation over a significant period, we feel this is more likely to be due to changes in uncontrolled parameters

### Process End-Point Detection from MSPC Charts

**Fig. 9:**
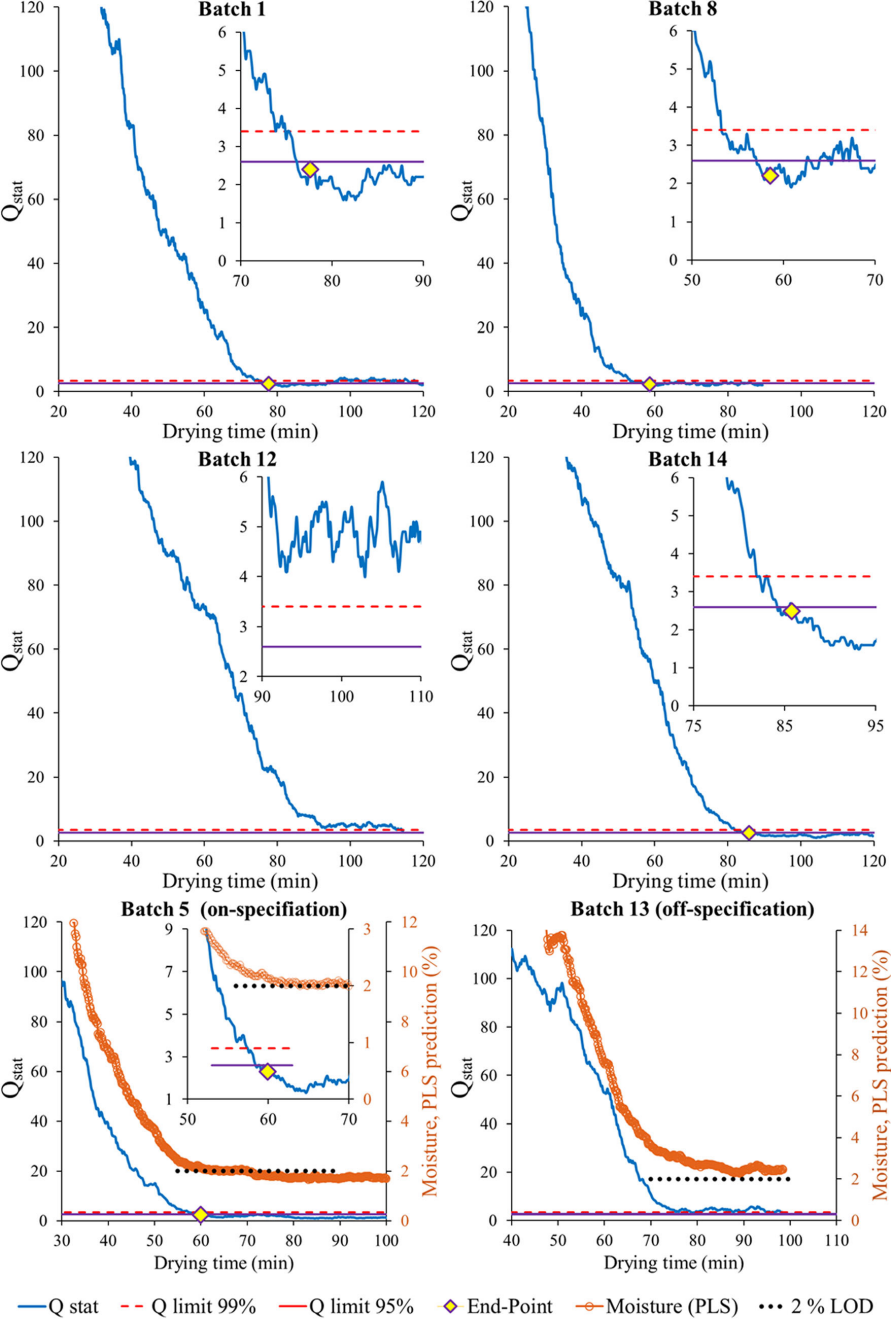
MSPC control charts for batches 1, 5, 8 and 14 (on-specification), 12 and 13 (off-specification). Inserted figures show in detail the final time range of the drying process and the process end-point, identified when 10 consecutive observations of Q _stat_ values were below the 95% control limit. Batches 5 and 13 include the moisture predictions from PLS model for reference (secondary axis).

A different application to evaluate the performance of the MEMS-FPI is the detection of the process end-point from the NIR signal. We use the MSPC model (Eq. 2) based on the end-point spectra from the calibration batches. This model required two principal components, PC1 marks the inverted water band and PC2, with a less interpretable shape, is required for the description of batch-to-batch variability (the PC loadings and related description are provided in the supplementary information S4). *Q*_*stat*_ control charts were calculated from Eqs. 2–4 for validation batches by projecting NIR spectral observations (using same pre-processing procedure as before) onto the developed model. Figure 9 shows the *Q*_*stat*_ MSPC charts obtained for six validation batches. Detected end-points are indicated with a yellow diamond marker in the *Q*_*stat*_ control charts. Batches 1, 5, 8 and 14 reached a final moisture content below 2% (on-specification), batches 12 and 13 did not (off-specification). For comparison, moisture content levels from the NIR spectra were compared in the MSPC control charts for batches 5 and 13 (moisture axis is at the right of the plot). These plots in Fig. 9 show that based on moisture levels the endpoint would have been delayed for batch 5; both *Q*_*stat*_ and the moisture level agree that batch 13 is off specification.

The results in Fig. 9 also confirm that a lower gas flow rate (batches12–14, see Table I) significantly increases the end-point process time (compared with on-specification batches, 1, 5 and 8). Similar results were observed for the other validation batches (see Supplementary Material). The spectral quality of the NIR sensor and its robustness is clearly sufficient for the construction and application of PCA-based MSPC models. The device has clear potential in end-point detection applications, promising a significant reduction in offline moisture measurements.

### Process Monitoring (Mass Transfer Resistance)

**Fig. 10:**
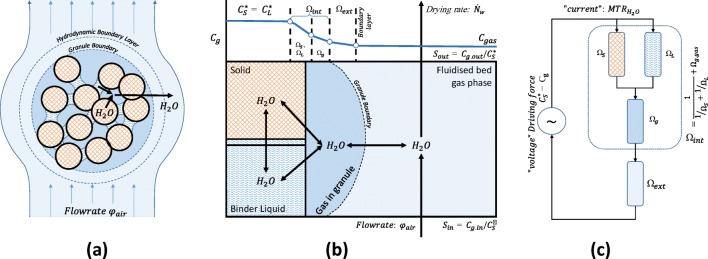
**(a)** a granule consists of solid held together by liquid bridges formed by the binder fluid. Water is also absorbed by solids (~0.3 g_water_/g_solid_). **(b)** The process scheme shows the location of water in different environments (“phases”) with arrows representing mass transfer between the environments. The gas phase concentration over (in equilibrium with) the solid (${C}_S^{\ast }$) and liquid (${C}_L^{\ast }$) are assumed to be similar. **(c)** The mass transfer may be represented as a resistance model with a “current” of water ($MT{R}_{H_2O},\frac{mol}{s.{m}_{bed}^3}$) flowing from high to low concentration. The transport through each environment requires a fall in concentration that is proportional to the “current”: $\Delta C= MT{R}_{H_2O}\times \Omega$, where Ω is the so called mass transfer resistance. The driving force (the “voltage”, the sum of all Δ*C*) equals the concentration difference between the source of the water and the final sink, the fluidising gas: ${C}_S^{\ast }-{C}_g$.

The promise of low cost NIR devices lies in the common availability of online compositional analysis. Our statistical analysis (PLS and MSPC) demonstrates the low cost MEMS-FPI NIR sensor to be a device that is suitable for composition measurement, with sufficient performance compared to conventional sensors when applied to fluid bed systems. To date NIR data has not been used in conjunction with mechanistic drying models to provide scale up data. As can be seen in Fig. 4, such analysis is complex, and requires the fusion of data from multiple sensor, as well as an understanding of material properties such as water adsorption isotherms and the vapour pressure of water. Methods to determine the bed moisture content and drying rate from temperature and humidity data show a significant deviation from samples analysed by LOD (33). To demonstrate the utility and value of continuous composition data in process monitoring we developed a methodology to monitor the process by evaluating the mass transfer resistance(s) from the available data. These resistances underpin fluidised bed scale up calculations. An overview of the mass transfer model is given in Fig. 10.

#### Step 1: Obtain the Molar Drying Rate

**Fig. 11:**
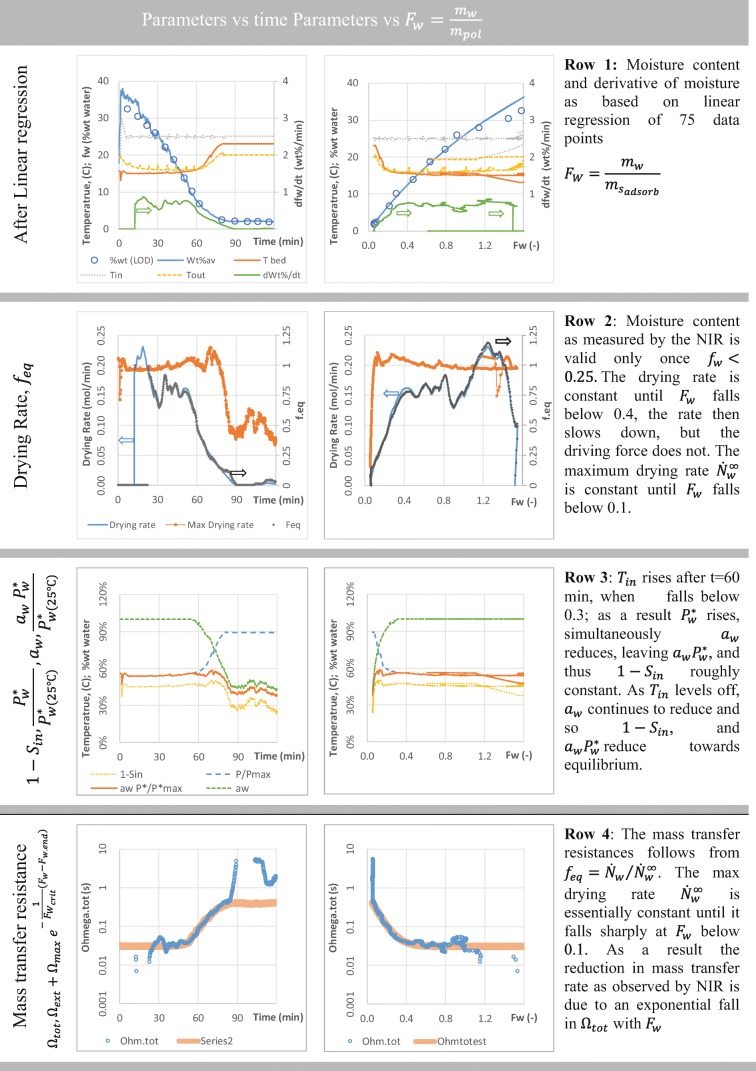
Determination of mass transfer resistances on experiment 14 (600 L/min).

To evaluate the mass transfer we first must convert the Drying curve *f*_*w*_(*t*), to the drying rate in mols/s by differentiation of the water content ${N}_w=\frac{1}{0.018}\ \frac{m_s\ {f}_w(t)}{1-{f}_w(t)\ }$ in the bed. The slope of the dying curve results from linear regression of a line to 36 data points either side of time t (a total of 72 points over 720 s). The standard error in the slope obtained is between 3%–5%. An example of *f*_*w*_, and its derivative *df*_*w*_/*dt* are shown in Fig. 11 row 1. The first graph shows the conventional drying curve consisting of the initial transient phase followed by the constant drying rate period (20–60 min, $\frac{d{f}_w}{dt}\approx 0.8\ wt\%/\mathit{\min}$) and the falling rate period and finally equilibrium (*f*_*w*_ ≈ 1.6 *wt* % ). Row 2 of the same figure shows the molar drying rate at approximately 0.15 mol/s in the constant drying rate regime.

#### Step 2 Work out the Driving Force

Granules consist of solids bound together by a binder fluid (Fig. 10a). Water is present in liquid bridges, but also adsorbed to some of the solid materials. The process scheme (Fig. 10b) shows the location of water in different environments (e.g. different phases and segregated domains) with arrows representing mass transfer between the environments. The mass transfer process is a diffusion process driven by the concentration gradient between water at the source in the granule and the water in the fluidising gas (the sink). As the liquid and solid phases in the granule are in intimate contact we assume equilibrium hence the gas phase concentration over (in equilibrium with) the solid (${C}_S^{\ast }$) and liquid (${C}_L^{\ast }$) are the same. As at some point the liquid phase will disappear, we focus our attention on obtaining ${C}_S^{\ast }$ as function of temperature and water content. Thermodynamically, the vapour pressure over a solid or liquid phase is represented by the product of the activity of water in that phase *a*_*w*_ and the vapour pressure of water at the bed temperature, ${P}_w^{*}\left({T}_{bed}\right)$:

7 $${C}_S^{\ast }=\frac{a_w{P}_w^{*}\left({T}_{bed}\right)}{R\ {T}_{bed}}\ \frac{mol}{m^3}$$

The equilibrium vapour pressure of pure water is given by the Antoine equation (34), supplementary material), here expressed relative to the vapour pressure at a reference temperature of 20 ° *C*:

8 $${P}_w^{*}\left(T\left({}^{\circ}C\right)\right)=2339.1\ast {e}^{-4078.8\ast \left(\frac{1}{236.63+T\left({}^{\circ}C\right)}-\frac{1}{256.63}\right)}\pm 1\ Pa$$

**Fig. 12:**
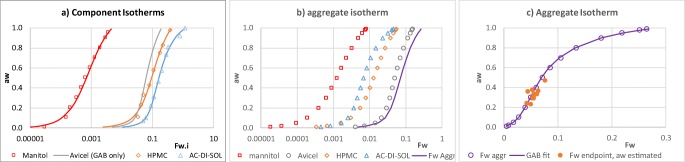
The construction of the aggregate isotherms: (**a**) Isotherms of individual components data is taken from the references listed table X, lines are the GAB equation fitted to the data using the parameters in Table X. (**b**) Contribution of the individual materials to the total water content F_W_ based on the mass of the polymers (mannitol weight not included) (**c**) The GAB fit to the aggregate isotherm. The estimated relative humidity plotted *versus* the moisture content of the final product measured by LOD corresponds well to the aggregate isotherm.

The water activity (*a*_*w*_) follows from the water adsorption isotherms of the materials present in the placebo.^2^ Such isotherms relate *a*_*w*_ to the water content on a *dry weight* bases (Fig. 12a). The GAB correlation, an extended BET equation developed by Guggenheim, Andersen and de Boer (35,36), expresses water content of solid *i*$\left({F}_{w_i},\mathrm{dry}\ \mathrm{basis}\right)$ as function of the water activity (Table II, and supplementary material):

9 $${F}_{w_i}\left({a}_w\right)=\frac{m_{w_i}}{m_{s_i}}=\frac{{k_w}_i{a}_w{C}_{GA{B}_i}{m}_{o_i}}{\left(1-{k}_{w_i}{a}_w\right)\left(1+\left({C}_{GA{B}_i}-1\right){k}_{w_i}{a}_w\right)}$$

**Table II:** Fitting Parameters for the GAB Equation of the Materials in the Placebo Formulation

Material	mass (gr)	${m}_{o_i}$	${C}_{GA{B}_i}$	*k*_*wi*_,	Reference
Mannitol	213	0.00068	1.73	0.87	Data (37)
Avicel PH-101	96	0.040	17.4	0.80	GAB param (38)
Hypromellose 2910	17	0.018	18.9	0.99	Data (39)
AC-Di-Sol	5	0.095	13.4	0.92	Data (40)
Aggregate	118	0.0441	14.30	0.846	Mass weighted average

At the beginning of the drying the Hypromellose 2910 (5 wt% dry basis) and Ac-Di-Sol (1.5 wt%) contain up to 50% of the adsorbed water with the remainder adsorbed onto the Avicel (29 wt%). As the granule dries, this reduces to ~10% and most of the remaining water is associated with the Avicel. Assuming the materials do not interact, an aggregate isotherm may be constructed by combining the water content adsorbed by the various materials at the same water activity (Fig. 12b). The aggregate isotherm correlates the granule’s water activity to *F*_*w*_ = *m*_*w*_/*m*_*adsorb*_, where *m*_*adsorb*_ is the combined mass of Avicel, Hypromellose and Ac-Di-Sol (Fig. 12c, fitted GAB parameters in Table 22).

#### Step 3: Link the Molar Drying Rate and the Driving Force

The mass transfer process can be represented with a resistance model ((41), Fig. 10c) that sees a “current” of water ($MT{R}_w,\frac{mol}{s.{m}_{bed}^3}$) flowing from the high concentration at the source (liquid bridges, solids) to low concentration in the gas used to fluidised the bed. The transport through each environment requires a fall in concentration that is proportional to the “current”,Δ*C*_*i*_ = *MTR*_*w*_ × *Ω*_*i*_, where *Ω*_*i*_ is the a called a *mass transfer resistance* which has the units of seconds. The overall driving force (equivalent to “voltage”) is the sum of all these Δ*C*_*i*_ and equals the concentration difference between the source of the water and its final sink, the fluidising gas:

10 $${C}_S^{\ast }-{C}_g=\sum \Delta {C}_i= MT{R}_{w}\times \sum {\varOmega}_i$$

The above equation shows the total mass transfer resistance *Ω*_*tot*_ =  ∑ *Ω*_*i*_ to be the sum of the individual resistances, in similarity with Ohm’s law. As the residence time of the gas is short (< 100 ms) it is common in most fluidised bed models to assume that the mass transfer resistance Ω_*tot*_ and the bed’s temperature and moisture content are constant on the time scale required for the gas to flow from the bottom to the top of the bed. A mass balance over a horizontal slice of the bed with volume *dV*_*bed*_ requires the gain of water in the gas flow (*φ*_*g*_ *dC*_*g*_) to be equal to the mass transferred from the granules to the air (*MTR*_*w*_ *dV*_*bed*_):

11 $${\varphi}_g\ d{C}_g={MTR}_w\ d{V}_{bed}={MTR}_w\frac{1}{f_s{\rho}_s}d{m}_s$$

Here *f*_*s*_is the volume fraction solids in the fluidised bed (estimated at 40%), and the *ρ*_*s*_ solids skeletal density (averaged at 1500 kg/m^3^). Substitution of Eq. 7 in 11 and integration yields (see supplementary material):

12 $${C}_{g. out}-{C}_{g. in}={f}_{MTR}\ \left(1-{S}_{in}\right)\ {\mathrm{C}}_S^{*}\kern0.75em with\kern0.5em {f}_{MTR}=\left(1-{e}^ {\large -\frac{m_s}{\Omega_{tot}{f}_s{\rho}_s{\varphi}_g}} \right)$$

Here ${S}_{in}={C}_{g. in}/{C}_S^{\ast }$ is the degree of saturation of the inlet gas which varies between 0 (no water) to 1 for an inlet gas in equilibrium with the water in the granules. It is important to realise that the saturation of the inlet gas (*S*_*in*_) may change during processing, as ${C}_S^{\ast }$ varies with both bed temperature and water content. We estimated *C*_*g*. *in*_ such that the outlet air is saturated at the beginning of the constant drying rate period.

The molar drying rate ${\dot{N}}_w$ in the fluid bed dryer now follows from the air flowrate *φ*_*g*_ and the concentration change calculated from Eq. 12:

13 $${\dot{N}}_w=\left({C}_{g. out}-{C}_{g. in}\right)\ {\varphi}_g={f}_{MTR}\kern0.5em {\dot{N}}_w^{\infty}\kern0.75em \mathrm{with}\ {\dot{N}}_w^{\infty }=\left(1-{S}_{in}\right)\ {C}_S^{\ast }\ {\varphi}_g\kern0.5em$$

*f*_*MTR*_ is the extent to which mass transfer is limiting: when *f*_*MTR*_ = 0 the mass transfer resistances are high and no significant transfer occurs. If on the other hand *f*_*MTR*_ = 1 then mass transfer is instantaneous, and the gas phase leaves saturated resulting in the maximum drying rate ${\dot{N}}_w^{\infty }$. The 2rd row in Fig. 11 shows the molar and maximum drying rates. The drying rate is about 80% of the maximum drying rate in the constant rate period which ends at *F*_*w*_ ≈ 0.35, after which the rate reduces in a manner that appears proportional with *F*_*w*_. Conversely, the maximum drying rate remains stable at *F*_*w*_ < 0.35, as *T*_*bed*_ and ${P}_W^{*}\left({T}_{bed}\right)$ increase balanced by a reduction in the water activity *a*_*w*_ as water is removed. The reduction of *a*_*w*_ becomes dominate when *F*_*w*_ < 0.1 the driving force and drying rates reduce then sharply.

#### Step 4 Calculate the Overall Mass Transfer Resistance

The mass transfer resistance Ω_*tot*_ follows from the ratio of the molar drying rate ${\dot{N}}_w$ measured by NIR, and the maximum drying rate ${\dot{N}}_w^{\infty }$ that follows from the bed temperature and the gas flowrate:

14 $${f}_{MTR}=\frac{{\dot{N}}_w}{{\dot{N}}_w^{\infty }}=\frac{{\dot{N}}_w}{\left(1-{S}_{in}\right)\ {C}_S^{\ast }\ {\varphi}_g}=1-{e}^{\large -\frac{m_s}{\Omega_{tot}{f}_s{\rho}_s{\varphi}_g}}$$

The measured temperature and water content data combined with the aggregate isotherm and the vapour pressure of water allows calculation of *f*_*MTR*_. The overall mass transfer resistance then follows by rearranging

15 $${\Omega}_{tot}=-\frac{m_s}{Ln\left(1-{f}_{MTR}\right)\ {f}_s{\rho}_s{\varphi}_g}$$

**Fig. 13:**
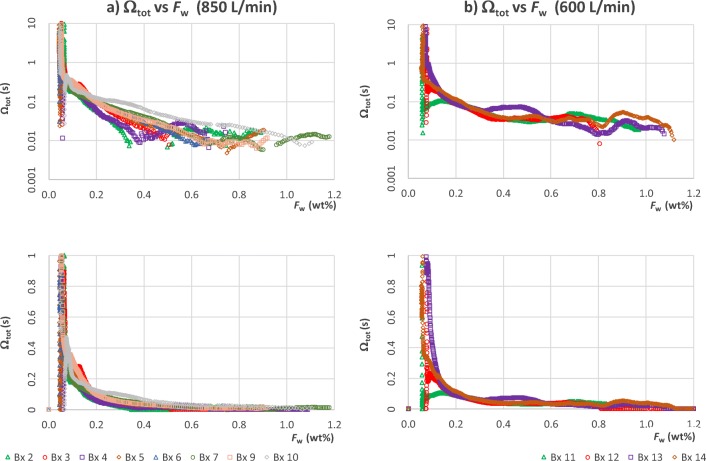
Mass transfer resistance curves for repeat batches; top) logarithmic Y axis, bottom) linear Y axis. The data demonstrates that at air flowrate of 850 L/min (**a**) the initial and final mass transfer resistance are relatively constant, but the internal resistance starts to dominate at widely different moisture content *F*_*w*_ = *m*_*w*_/*m*_*adsorb*_. At 600 L/min (**b**) we observe more consistent mass transfer resistance trajectories.

This is shown in row 4 of Fig. 11. After the steady state is reached, Ω_*tot*_ ≈ 0.03*s*, but once *F*_*w*_ drops below 0.4, the mass transfer resistance starts to increase, eventually it is an order of magnitude higher. This behaviour is observed in all batches (Fig. 13). At 600 L/min, the curves of the different repeat batches are consistent, even though the 20% relative error in moisture content level does result in significant fluctuations around the mean. The increase in the internal resistance by a factor 30 to 70 is clearly visible in all batches displayed bar Bx 11.

#### Step 5 Evaluation of the Observed Mass Transfer Resistance

To parametrise, the observed mass transfer resistances we based on a constant resistance external to the granule, and an internal granule resistance that falls exponentially with moisture content:

16 $${\Omega}_{tot}={\Omega}_{ext}+{\Omega}_{max}\ {e}^{-\frac{1}{F_{W_{crit}}}\left({F}_w-{F}_{w. end}\right)}$$

**Fig. 14:**
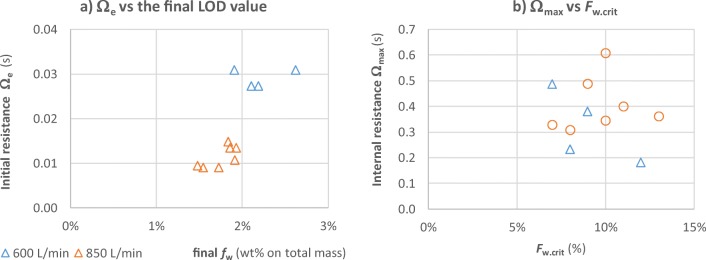
Mass transfer resistance parameters (**a**) the external resistance Ω_*e*_ reduces with increasing flowrate, (**b**) the internal resistance increases exponentially with water content *F*_*w*_ = *m*_*w*_/*m*_*absorb*_. The resistance at the end of drying, Ω_*max*_, is an order of magnitude higher than the initial external resistance Ω_*e*_.

Here *F*_*w*. *end*_ is the final moisture content relative to *m*_*adsorb*_. Table III and Fig. 14 shows these parameters for the experiments conducted. As expected, the external resistance varies with airflow reducing from 0.028 *s* ± 15% at 600 L/min, to 0.013 s±27% for 850 L/min. The maximum resistance (0.64 ±33 % ) and the critical moisture content ${F}_{w_{crit}}$ (0.10 ± 27 % ) appears to be independent of the flowrate. The external resistance will dominate at moisture contents above *F*_*w*_ = 0.5 as only *e*^−(0.5 − 0.1)/0.1^ ≈ 2% of the internal resistance remains (~0.014 s).

**Table III:** Average Mass Transfer Resistances for the Placebo Granules

Flowrate	600 L/min	850 L/min	All
Data points	4	8	12
Ω_*ext*_ (*s*)	0.028 ± 15%	0.013 ± 27%	
Ω_*max*_ (*s*)	0.58 ± 33%	0.68 ± 34%	0.64 ± 33%
${F}_{W_{crit}}\ \left(-\right)$	0.10 ± 20%	0.10 ± 31%	0.10 ± 27%

The obtained mass transfer parameters are difficult to reconcile with literature, as generally only the drying rate is reported (33,42). It is worth noting that the variation in the estimated parameters related to mass transfer is double the error in the NIR based moisture content (±13%). Even so, the mass transfer analysis using low cost NIR sensors is able to detect the change from externally controlled mass transfer (the so called constant rate period) to mass transfer limited by the internal resistance of the granule. The rate of change of the internal resistance is exponential, which is inconsistent with a shrinking core model in which the volume of the granule that contains water shrinks towards the core of the granule (43). Further work with controlled humidity will be required to see if the analysis is robust.

A detailed phenomenological interpretation of the presented results is beyond the scope of this paper, but it appears that the desorption kinetics dominate mass transfer once the binder liquid droplets have evaporated. It follows that the ingredient selection and adsorption characteristics can have a profound effect on the drying time required.

## Conclusions

A new reduced cost and small form factor MEMS FPI NIR sensor has been tested over a 9 month period during which 14 batches of placebo granules have been manufactured and dried in a fluidised bed. Overall, the MEMS-FPI sensor performance gave a very satisfactory stability and reproducibility and delivered high quality, continuous data robustly during fluid bed drying off placebo pharmaceutical granules; a typical processing scenario in which acquired NIR spectra have a very low signal to noise ratio. We tested the sensors performance with three applications: moisture monitoring, end-point detection and process analysis (mass transfer monitoring).

In fluidised beds abrupt changes in the spectra collected from scan to scan occur because of the random motion of the placebo granules. Using spectra averaged over 12 min, a satisfactory and robust PLS regression model was developed to predict the moisture content from NIR. The accuracy of the moisture content prediction over a significant number of batches and an experimental period of 3 months remained constant at a relative error of 13%. This is of a similar magnitude as reported for fluidised beds dryers using high specification commercial NIR spectrometers. The consistent performance demonstrates the NIR sensor potential for use as process sensor.

In the second application, NIR spectra collected were used to develop a MSPC model with a 2% endpoint moisture content target. This allowed successful endpoint detection, and correct identification of off-specification batches using only the NIR sensor’s data.

To demonstrate the utility and potential benefits of having cheap online sensors available for process monitoring, we fused temperature and NIR generated moisture data to determine the mass transfer resistance. Our analysis indicates that for the placebo granules the overall mass transfer resistance is the sum of a gasflow dependent external resistance (0.01–0.03 s) and a moisture content dependent internal resistance that increases exponentially as moisture content reduces (0.0 to 0.7 s). We demonstrated that this low cost NIR sensor allows the detection of changes in the drying mechanisms, which may give an early warning if unspecified physico-chemical properties of input materials (such as water adsorption isotherms) have changed.

In summary, the small form factor MEMS-FPI sensors has been shown to be a robust alternative for process monitoring. It is robust to vibration and temperature changes and straightforward to install. Its NIR spectra are of a sufficient quality to deliver composition related predictions with the same accuracy as commercial spectrometers in a system with an extremely low signal to noise ratio. The MEMS chip spectrometer can be mass produced and has a small enough form factor to be integrated in the next generation of plant sensors. Besides, it is cheap enough to allow multi point composition sensing in the way that is done to day for temperature, pressure and flow rate measurement.

## Electronic Supplementary Material

ESM 1 (DOCX 3228 kb)

ESM 2 (XLXS 704 kb)

## Nomenclature

*a*_*w*_: activity of water in the solid phase (−)
*b*, *b*_*o*_: regression coefficients
*C*_*g*_: Water conc. in the gas phase (*mol*/*m*^3^)
${C}_L^{\ast }$: Gas phase conc. in equilibrium with the liquid in the granules (*mol*/*m*^3^)
${C}_S^{\ast }$: Gas phase conc. in equilibrium with the water in the solid phase (*mol*/*m*^3^)
**E**: Matrix with residual errors of the PCA model (−)
**e**_***y***_: Error between measured and predicted values of y (−)
*e*_*rel*_: Relative error (−)
*f*_*s*_: Volume fraction solids in the fluidised bed (−)
*f*_*MTR*_: Extent to which mass transfer is limiting (−)
*f*_*w*_: Moisture fraction (wt% of total mass)
${F}_{w}$: Moisture fraction (wt% of dry solids)
*m*_*w*_, *m*_*s*_: Granule’s mass of water and solids respectively (*kg*)
*m*_*adsorb*_: Mass of water absorbing components in the granule (*kg*)
*MTR*_*w*_: Mass transfer rate(*mol*/*s*.*m*^3^)
*N*_*w*_: Number of moles of water (*mol*)
${\dot{N}}_w^{\infty }$: Maximum drying rate (*mol*/*s*)
${\dot{N}}_w$: Molar drying rate (*mol*/*s*)
**P**: Loadings matrix (−)
${P}_w^{\ast }$: Vapour pressure of water (*Pa*)
*Q*_*stat*_: Q-statistic (−)
*S*_*in*_: Degree of saturation of the inlet gas (−)
**T**: Scores matrix (−)
*T*_*bed*_: Bed temperature (*K*, or °*C*)
*V*_*bed*_: Volume of the fluidised bed (*m*^3^)
**y**: Response vector (−)
**X**: Matrix containing one spectrum on each row (−)
***x***_*new*_: Row vector containing 1spectrum (−)

## Greek Letters

*φ*_*g*_: Gas flowrate (*m*^3^/*s*)
*ρ*_*s*_: Fluid bed density (*kg*/*m*^3^)
*Ω*: Mass transfer resistance (1/*s*)
Ω_*ext*_, Ω_*max*_: Fitting parameters (1/*s*)
*Ω*_*tot*_: Overall or total mass transfer resistance (1/*s*)

## Footnotes

1 Note we adhere to the standard notation for chemometics, using *row* vectors ***r =*** (1, 2, 3) for data over a series of wavelengths. For row vectors the order of multiplication is reversed. So for ***r*** = ***v***^*T*^ if ${\boldsymbol{v}}_{\mathbf{2}}=\boldsymbol{A}\ \boldsymbol{v}\ \mathrm{then}\ {\boldsymbol{v}}_{\mathbf{2}}^T={\boldsymbol{r}}_{\mathbf{2}}=\boldsymbol{r}\ {\boldsymbol{A}}^T$. Bold small letters refer to vectors, bold capitals to matrices.

2 We ignore the reduction in water activity due to the presence of solutes, predominantly Mannitol at ~ 1 mol/L, which reduce *a*_*w*_ from 1 to 0.98 by Raoult’s law.
