# Supplementary material for: Process Monitoring of Moisture Content and Mass Transfer Rate in a Fluidised Bed with a Low Cost Inline MEMS NIR Sensor
Source: Pharm Res. 2020 Apr 21;37(5):84. doi: 10.1007/s11095-020-02787-y (PMC7174278; doi:10.1007/s11095-020-02787-y)
Supplement: Supplementary file 1 — (DOCX 3228 kb) [file 11095_2020_2787_MOESM1_ESM.docx]

**Process monitoring of moisture content and mass transfer rate in a fluidised bed with a low cost inline MEMS NIR sensor.**

**Authors:**

Claudio Avila ^†^, Joan Ferré ^§^, Rodrigo Rocha de Oliveira ^∥^, Anna de Juan Capdevila ^∥^,
Wayne Sinclair ^‡^, Faiz Mahdi ^†^, Ali Hassanpour ^†^, Timothy N. Hunter ^†^, Richard A. Bourne ^†^ and Frans L. Muller ^*†^.

**Affiliation and Address:**

^†^ School of Chemical and Process Engineering, University of Leeds, Leeds LS2 9JT, United Kingdom

^§^ Department of Analytical Chemistry and Organic Chemistry, Universitat Rovira i Virgili, Tarragona 43007, Spain

^∥^ Department of Chemical Engineering and Analytical Chemistry, Universitat de Barcelona, Barcelona 08028, Spain

^‡^ GlaxoSmithKline, Stevenage, United Kingdom

* Corresponding author

**Author email address:**

| Claudio Avila: C.R.Avila@leeds.ac.uk  Joan Ferré: joan.ferre@urv.cat  Rodrigo Rocha de Oliveira: rrochade10@alumnes.ub.edu  Anna de Juan Capdevila: anna.dejuan@ub.edu  Wayne Sinclair: wayne.e.sinclair@gsk.com | Faiz Mahdi: F.M.Mahdi@leeds.ac.uk  Ali Hassanpour: A.Hassanpour@leeds.ac.uk  Timothy N. Hunter: T.N.Hunter@leeds.ac.uk  Richard A. Bourne: R.A.Bourne@leeds.ac.uk  Frans L. Muller: F.L.Muller@leeds.ac.uk |
| --- | --- |

**Purpose:** The current trend for continuous drug product manufacturing requires new, affordable process analytical techniques (PAT) to ensure control of processing. This work evaluates whether property models based on spectral data from recent Fabry–Pérot Interferometer based NIR sensors can generate a high-resolution moisture signal suitable for process control.

**Methods**: Spectral data and offline moisture content were recorded for 14 fluid bed dryer batches of pharmaceutical granules. A PLS moisture model was constructed resulting in a high resolution moisture signal, used to demonstrate (i) endpoint determination and (ii) evaluation of mass transfer performance.

**Results**: The sensors appear robust with respect to vibration and ambient temperature changes, and the accuracy of water content predictions ($\pm13\%)$ is similar to those reported for high specification NIR sensors. Fusion of temperature and moisture content signal allowed monitoring of water transport rates in the fluidised bed and highlighted the importance water transport within the solid phase at low moisture levels. The NIR data was also successfully used with PCA-based MSPC models for endpoint detection.

**Conclusion**: The spectral quality of the small form factor NIR sensor and its robustness is clearly sufficient for the construction and application of PLS models as well as PCA-based MSPC moisture models. The resulting high resolution moisture content signal was successfully used for endpoint detection and monitoring the mass transfer rate.

**Keywords**

Near infrared spectroscopy; MEMS Fabry-Pérot Interferometer sensor; Fluidised bed drying; Mass transfer resistance, Online process monitoring

Table of Contents

[S1. Additional details on granules production and characterisation 2](#_Toc27737438)

[S2. Additional details on MEMS-FPI NIR sensor and data acquisition 6](#_Toc27737439)

[S3. Additional details on the prediction of moisture content using PLS model 7](#_Toc27737440)

[S4. Additional details on the prediction of the process end-point using MSPC models 10](#_Toc27737441)

[S5. Adsorbtion Isotherms 14](#_Toc27737442)

[S6. Water vapour pressure in pharma dryers 17](#_Toc27737443)

[S7. Derivation of Fluid bed Resistance model 21](#_Toc27737444)

[Acknowledgments 25](#_Toc27737445)

# S1. Additional details on granules production and characterisation

Pharmaceutical granules were produced in a high shear granulator using a wet granulation method (equipment described in paper) by mixing five powder components with water, which was sprayed at a constant liquid flow rate (5 - 6 ml min^-1^) up to reaching a desired water content of approximately 35%. During the granulation process, samples of granules (~5 g) were collected from the granulator bowl using a vacuum suction at specific process times (1, 10, 20, 30, 34, 38, and 42 min), to determine moisture content (using loss on drying, LOD), granule formation and particle size (using an image analysis method) at different process stages. In parallel, the granulation process was controlled online by measuring the torque profile and the temperature in the granulation bowl. The total granulation time was approximately 43-51 min. The step by step procedure followed for the fourteen batches is described below:

1. A total mass of 500 g of the dried components (approximately 1 litre in volume) were added into a clean and dry granulator bowl, and mixed for 60 s using an impeller speed of 800 rpm.
2. After the initial mixing, the impeller speed was kept constant to 800 rpm and 252 g of Reverse Osmosis Water (RO-Water) was sprayed on the mixed powders using an atomising nozzle at a feeding rate of 5-6 ml min^-1^.
3. During granulation, samples of approximately 5 g were retrieved at fixed time intervals (1, 10, 20, 30, 34, 38 and 42 min) for analysis.
4. When granules reached the desired moisture and size, the pump was turned off to stop the liquid addition.
5. The granulator continued mixing for an additional 60 s to ensure homogenous mixing of the liquid binder.
6. The final granules produced were stored in sealed containers, reducing the temperature from 55 ^o^C after granulation to 10 ^o^C and avoiding loss of moisture, before to be loaded into the fluidised bed dryer.

**Torque and moisture profiles during granulation**

Fig. ‎S2.1 shows the torque profile as a function of water addition during granulation for five batches of granules, with the same trend observed for the fourteen batches dried. The maximum torque value was around 2000 mNm which depended only on the solids components ratio (each component has specific solubility and hygroscopic properties). As this ratio was kept constant for all the fourteen batches, there was a good agreement between runs, which translated to good experimental reproducibility.


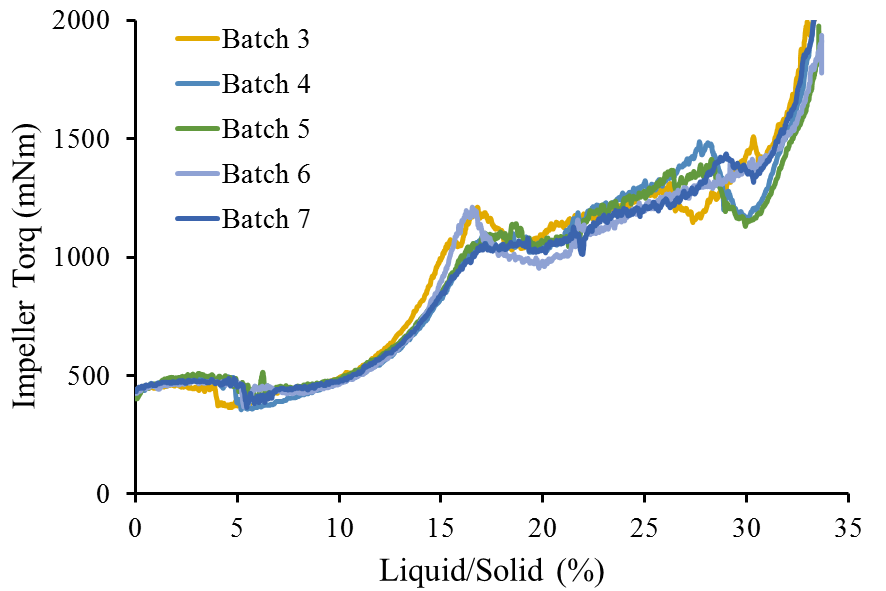


**Fig. S2.1.** Torque profiles observed during liquid addition through the granulation for five batches.

Fig. ‎S2.2 shows a comparison between the theoretical liquid mass balance and the analytical moisture measurements during granulation determined using a gravimetric method (LOD). These granule samples were collected using the inline sampling system at fixed time intervals (1, 10, 20, 30, 34, 38, and 42 min), and the difference between the theoretical and analytical measurement at the end of the process is due to the natural loss of moisture from the bowl (e.g. granules at 55 ^o^C releasing steam), which is normal and affected all batches.


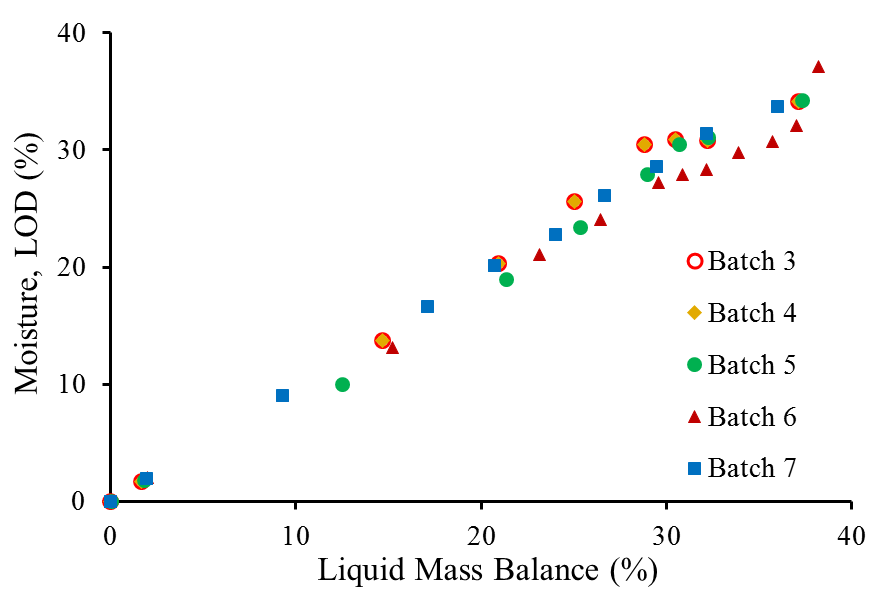


**Fig. S2.2**. Granule moisture profiles comparing the theoretical liquid mass balance and the analytical moisture measurements determined using loss on drying method. These results show consistency of the moisture levels during granulation for all runs.

**Granules size during granulation**

Fig. S2.3 shows SEM images obtained by using a desktop SEM instrument, from the samples collected during granulation using the same inline sampling system described (for a single batch). From these images, a link between the granule formation process with the solid/liquid ratio (during the continuous water spray varying from 2 to 38 % moisture level) was stablished. These images show the influence of the solid/liquid ratio on the size and shape of produced granules, and it was used as a guide to produce the fourteen batches required for drying.


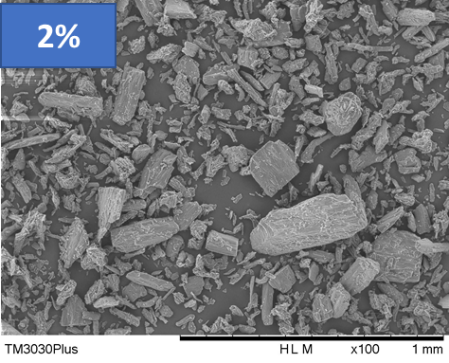

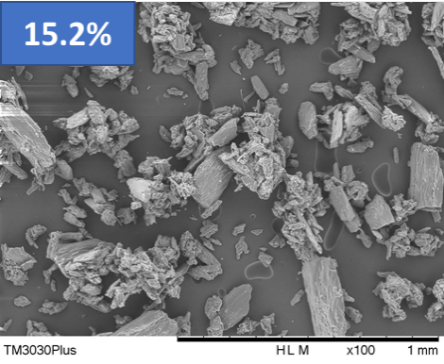

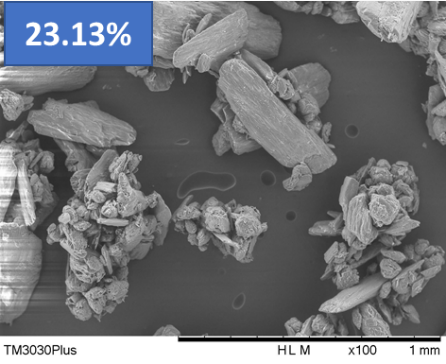

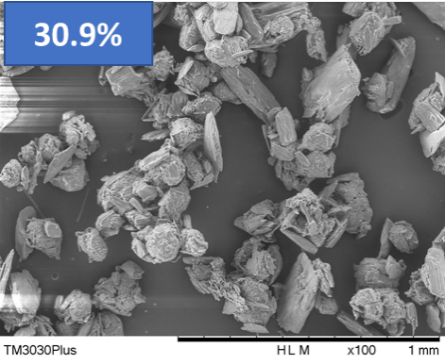

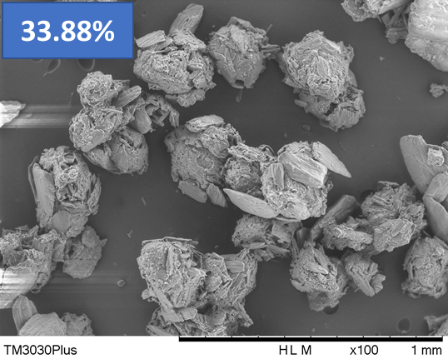

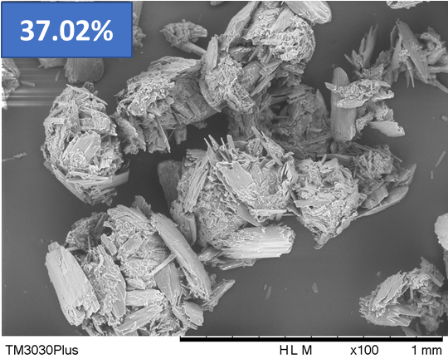

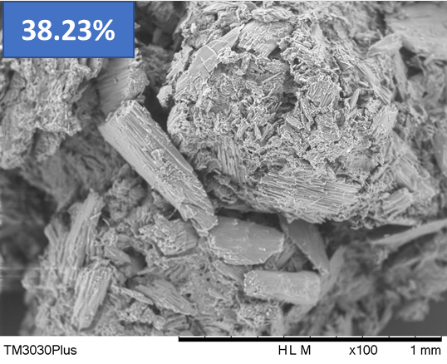


**Fig. S2.3.** Granules obtained at different stages of the granulation process, under an increasing moisture level during granulation (2% to 38.2 %). All images have the same magnification scale.

# S2. Additional details on MEMS-FPI NIR sensor and data acquisition

A novel spectral sensor model N-Series 2.2 by Spectral Engines (Finland) was used for the acquisition of the NIR spectra from 1750 nm to 2150 nm. A diagram of the sensor is shown in Figure 2 of the paper. The sensor has a single element extended InGaAs detector, with a tuneable MEMS Fabry-Pérot interferometer (FPI) filter acting as the spectral element. The sensor had an integrated light source model LS-PRO equipped with a miniature tungsten vacuum lamp as the illumination source.

The scanning mechanism implemented on these new sensors to construct the NIR spectra differs from the standard FTIR and grating systems conventionally employed. The FPI executes a scanning sequence where a voltage modulation adjusts the FPI filter gap, allowing only a particular light wavelength passing through the filter and sampled by the detector. After finishing, the FPI is readjusted to sample the next wavelength, subsequently repeating this action to cover the complete sensor wavelength range. The total time to obtain a single scan is a combination of: a) the time to scan the selected wavelength range. The positioning of the FPI is achieved in 1 ms, followed by the signal sampling for 0.1 ms. For instance, to scan the 400 nm range with a 1 nm wavelength step (experimental condition for the N-2.2 sensor used), the complete process takes 440 ms; and b) the time to transmit the signal from the sensor to the computer, which depends on the transmission speed of the communication protocol used. In this case the sensor was connected by USB 2.0 which takes approximately 500 ms (effective transmission time observed). As a result, the total time to complete and transmit a single scan was approximately 940 ms.

For all the experiments the energy output for the lamp was set to 50% of the maximum level. The sensor integration time was set to 0.1 ms and the wavelength step set to 1 nm (401 points obtained from the operational sensor range).

# S3. Additional details on the prediction of moisture content using PLS model

In addition to the details presented in Figure 5 (research paper), Figures S3.1 to S3.3 show the moisture content predictions from PLS model compared to the analytical moisture content determined using LOD, for the 14 batches performed.


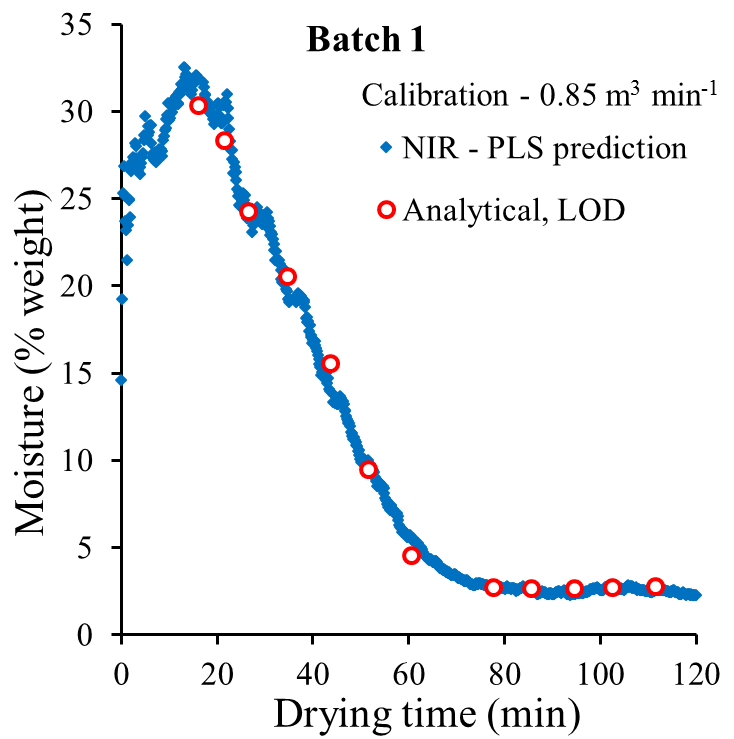

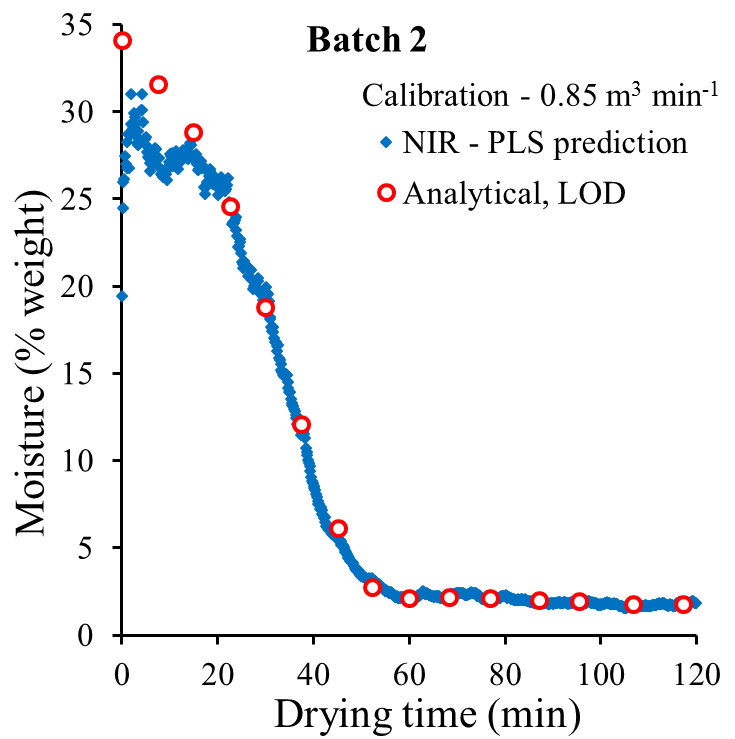

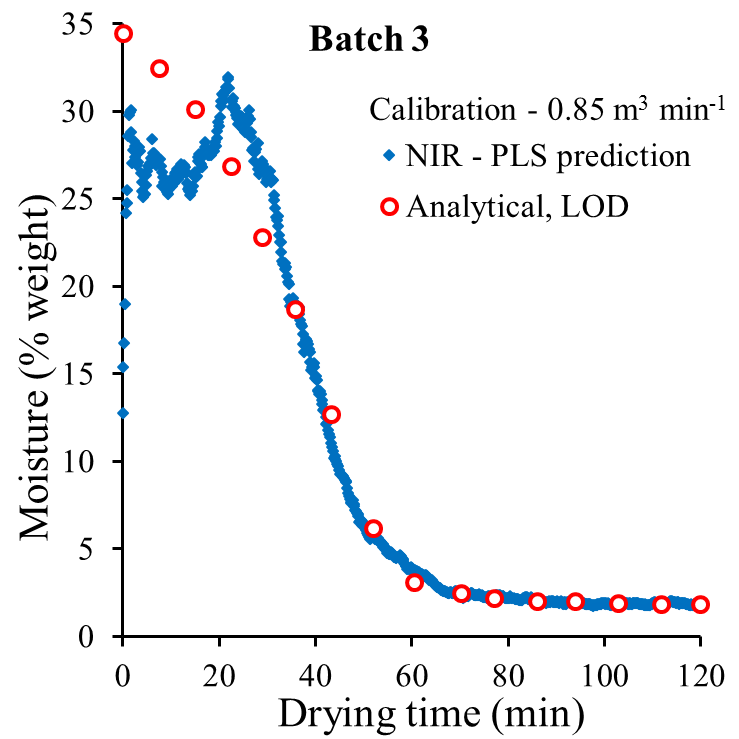

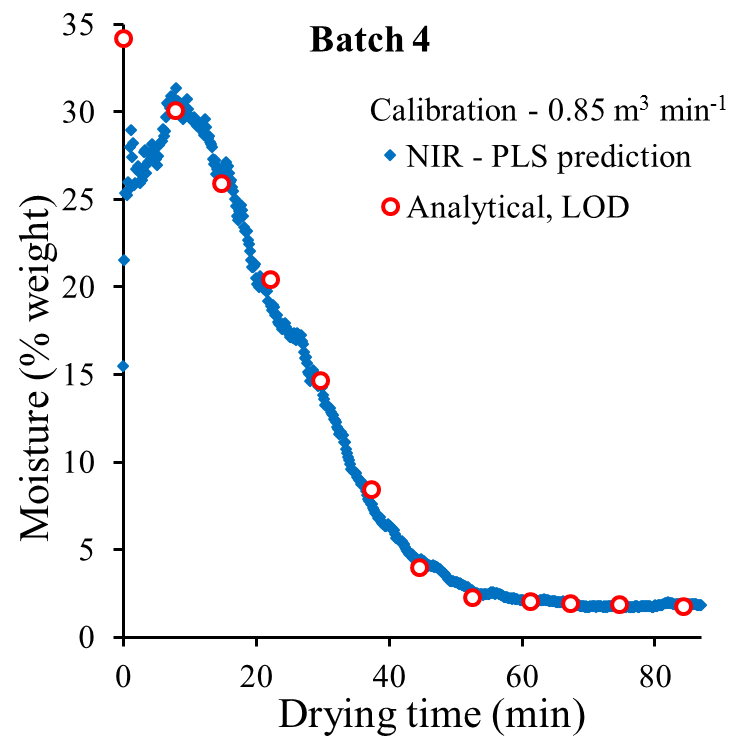


**Fig. S3.1.** Prediction profiles obtained from the PLS regression model using online NIR spectra (semi continuous line) compared to the analytical moisture content determined using LOD (discrete circles), for calibration batches 1, 2, 3 and 4 (using a fluidisation air flow rate of 0.85 m^3^ min^-1^).


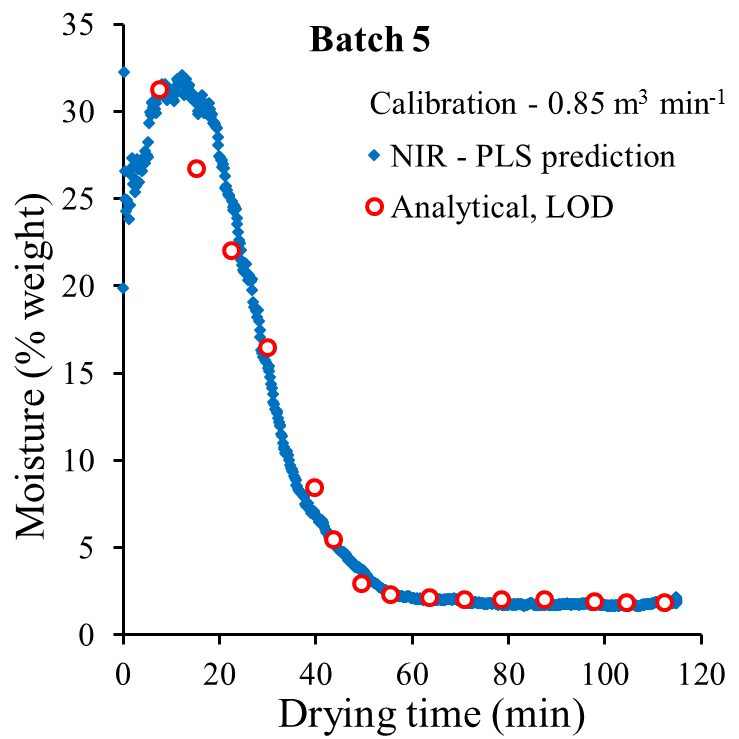

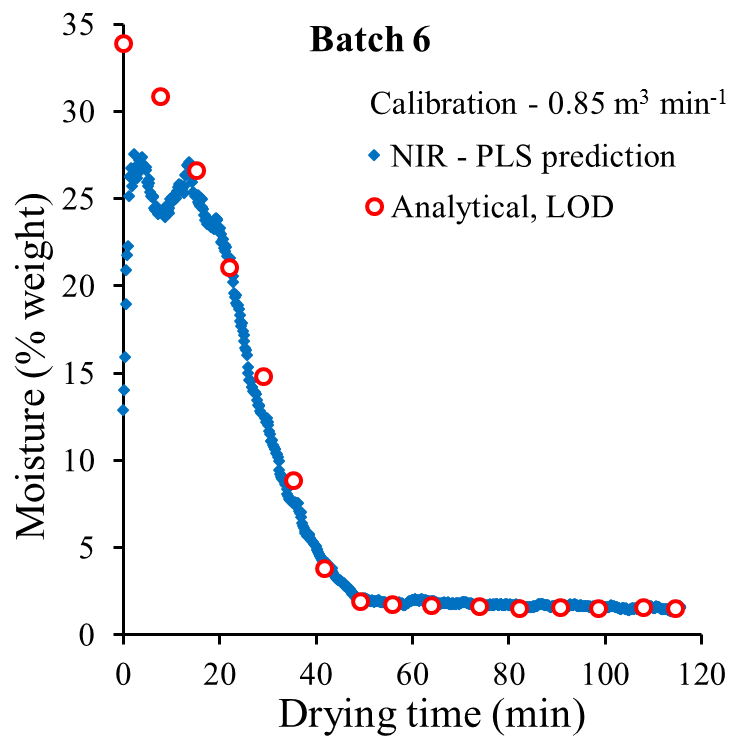


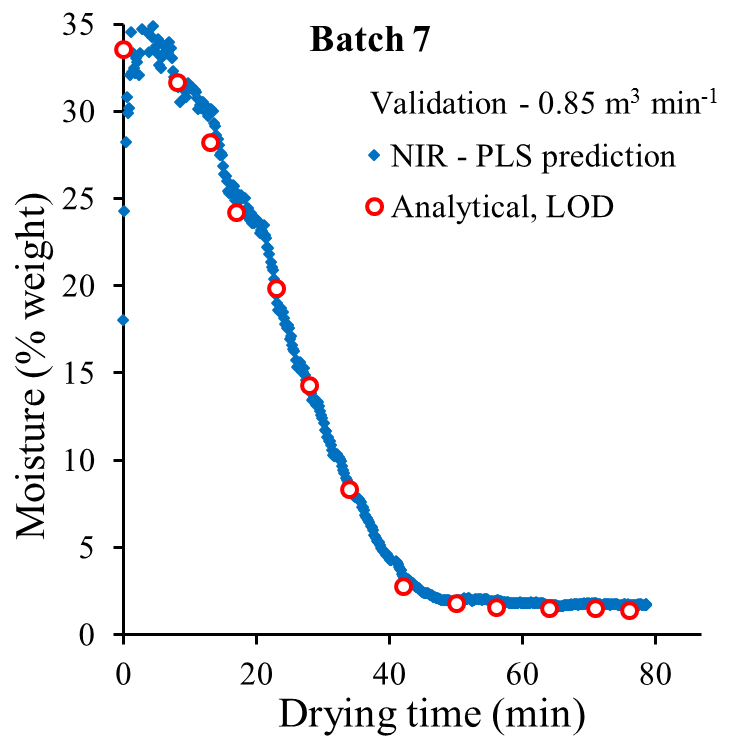

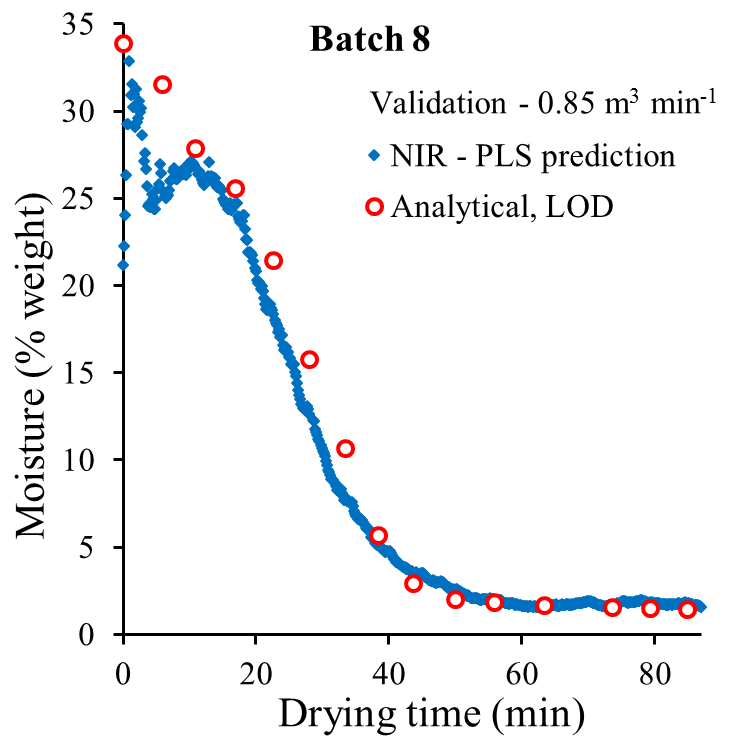


**Fig. S3.2.** Prediction profiles obtained from the PLS regression model using online NIR spectra (semi continuous line) compared to the analytical moisture content determined using LOD (discrete circles), for calibration batches 5 and 6, and validation batches 7 and 8 (using a fluidisation air flow rate of 0.85 m^3^ min^-1^).


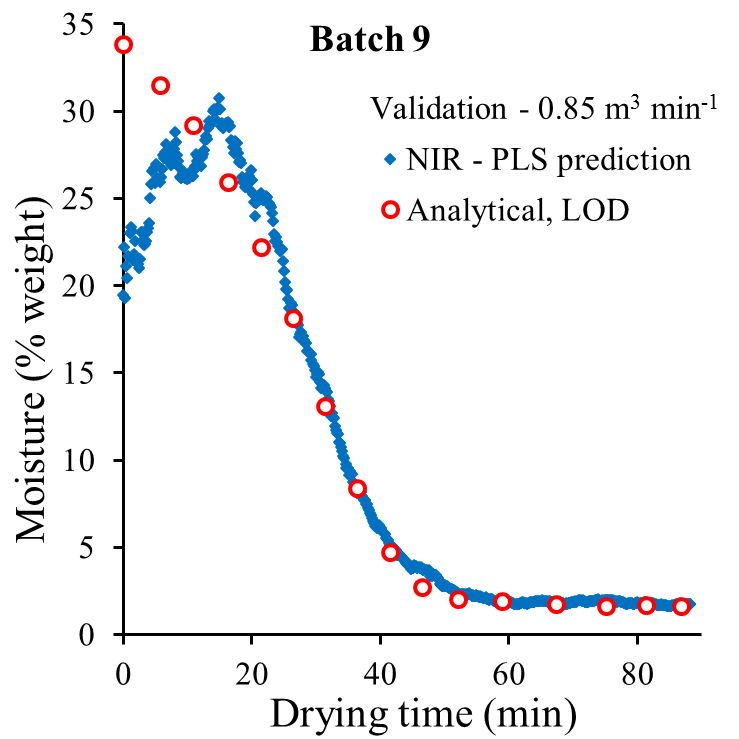

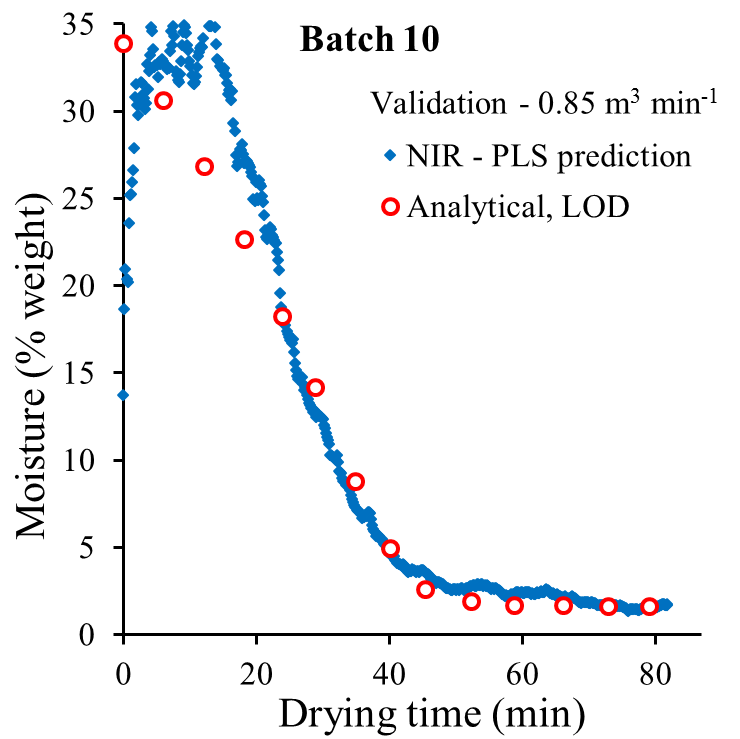

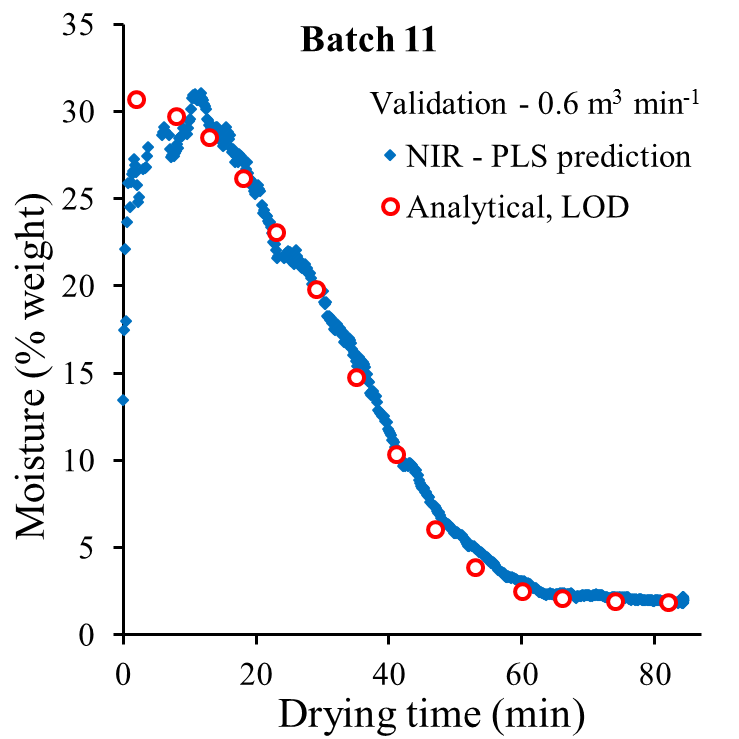

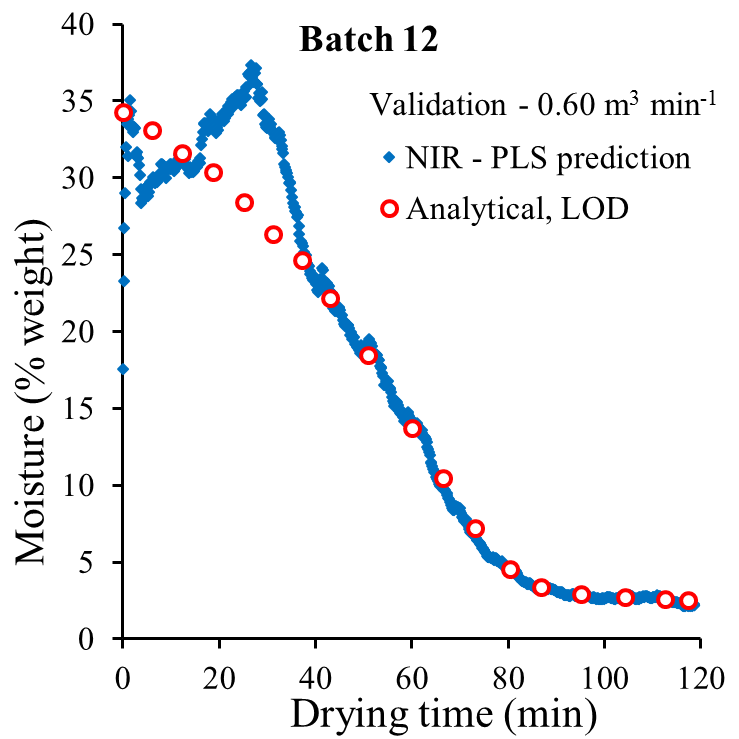

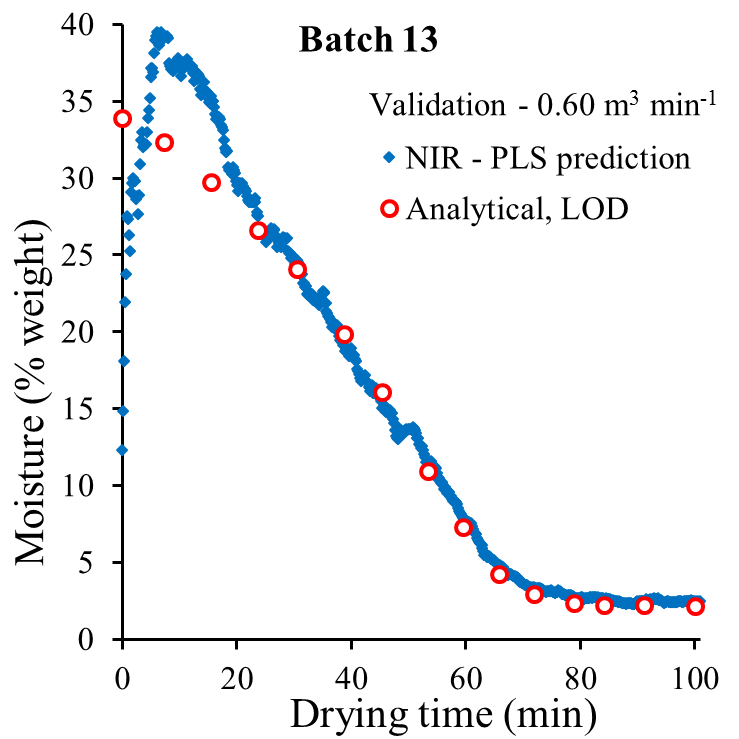

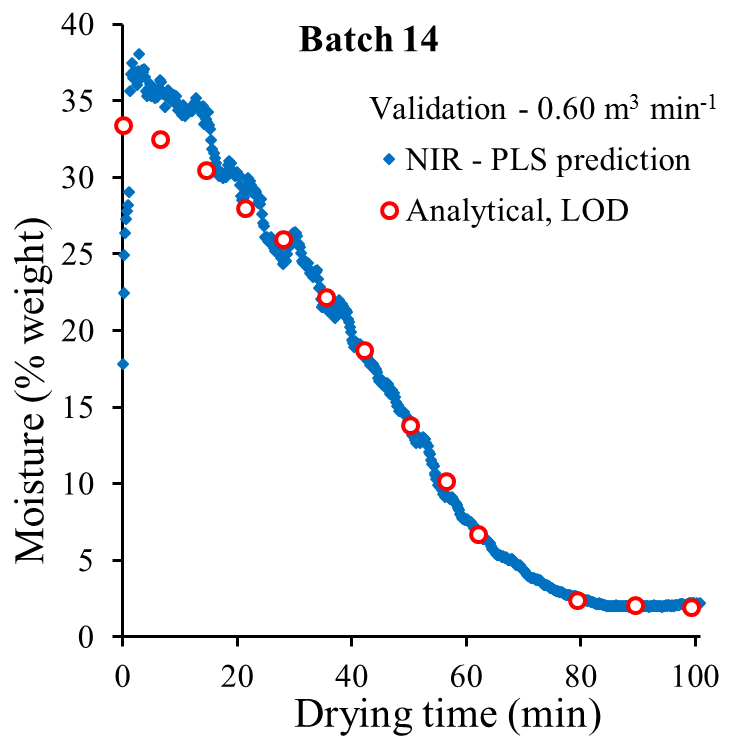


**Fig. S3.3.** Prediction profiles from the PLS regression model (semi continuous line) compared to analytical moisture content (LOD, discrete circles), for validation batches 9, 10 (fluidisation rate 0.85 m^3^ min^-1^), 11, 12, 13 and 14 (fluidisation rate 0.6 m^3^ min^-1^).

# S4. Additional details on the prediction of the process end-point using MSPC models

In addition to the details presented in Figure 7 (research paper), Figures S4.1 to S4.4 show MSPC control charts obtained for the 14 batches performed and Figure S4.5 shows the MSPC PCA loadings.


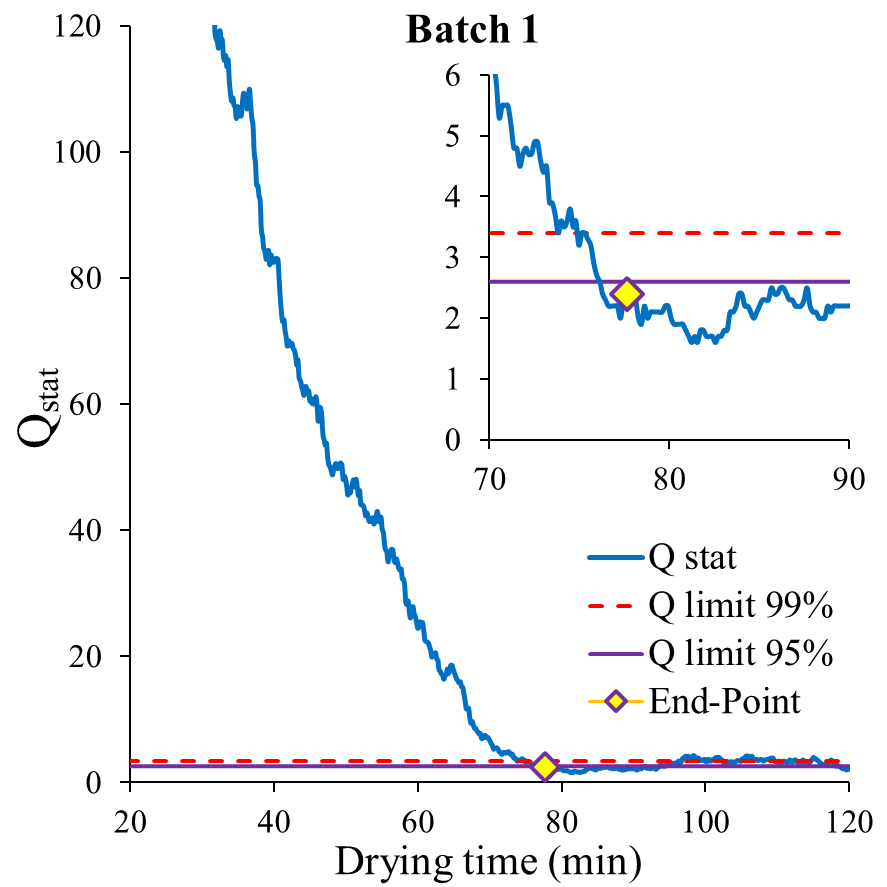

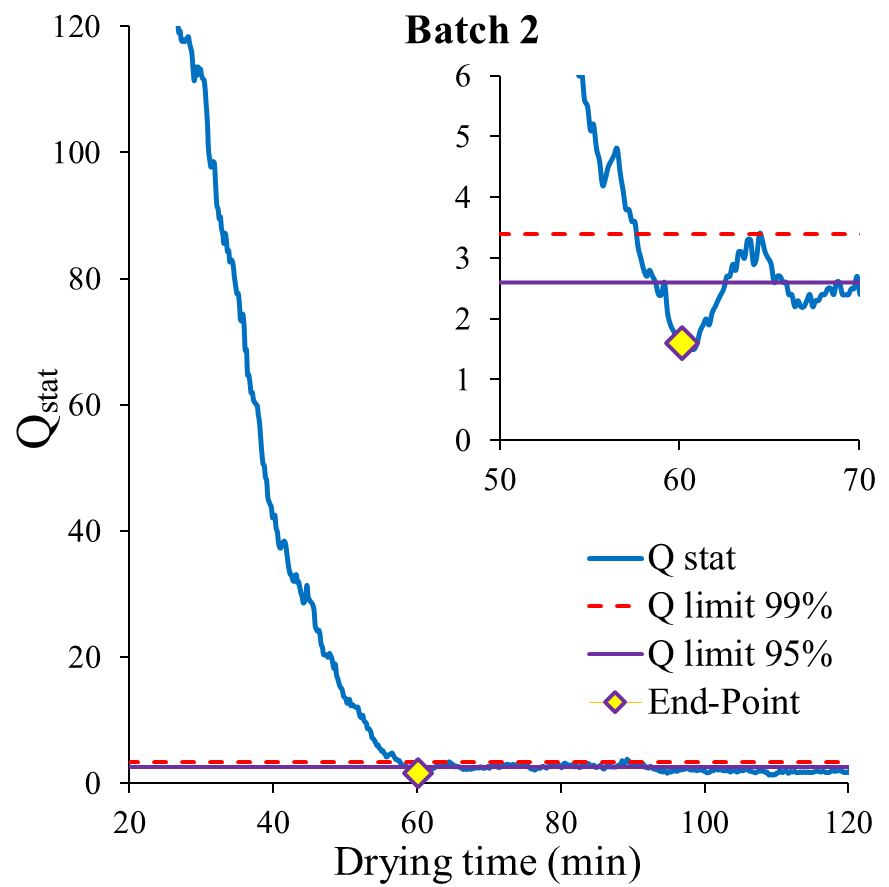

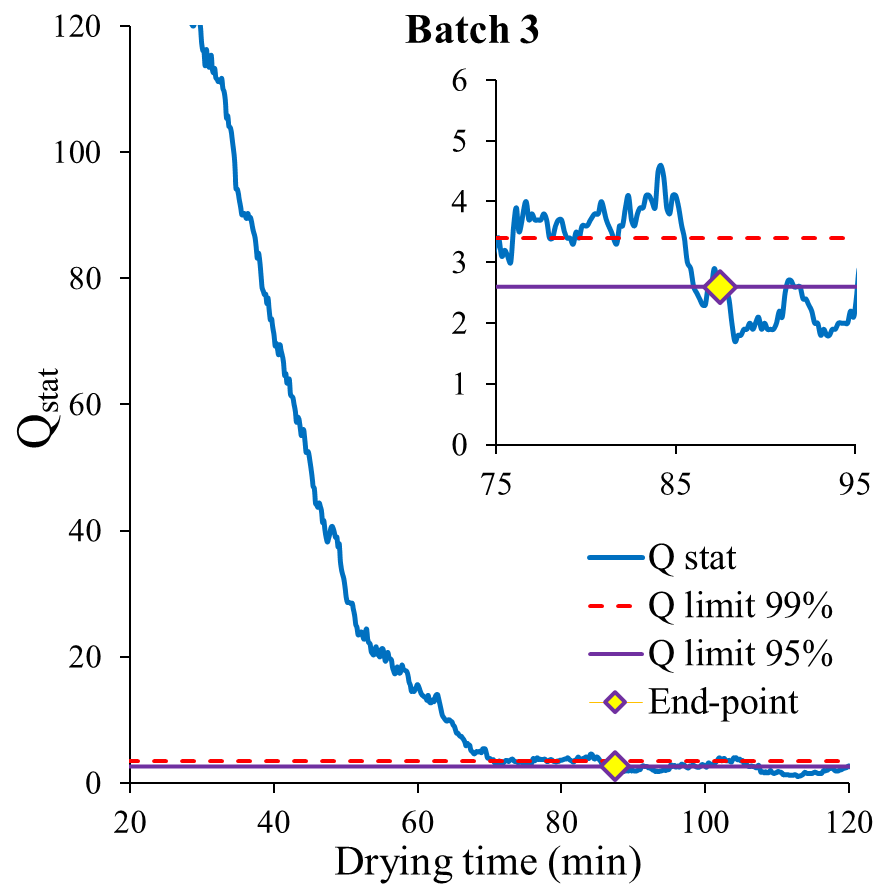

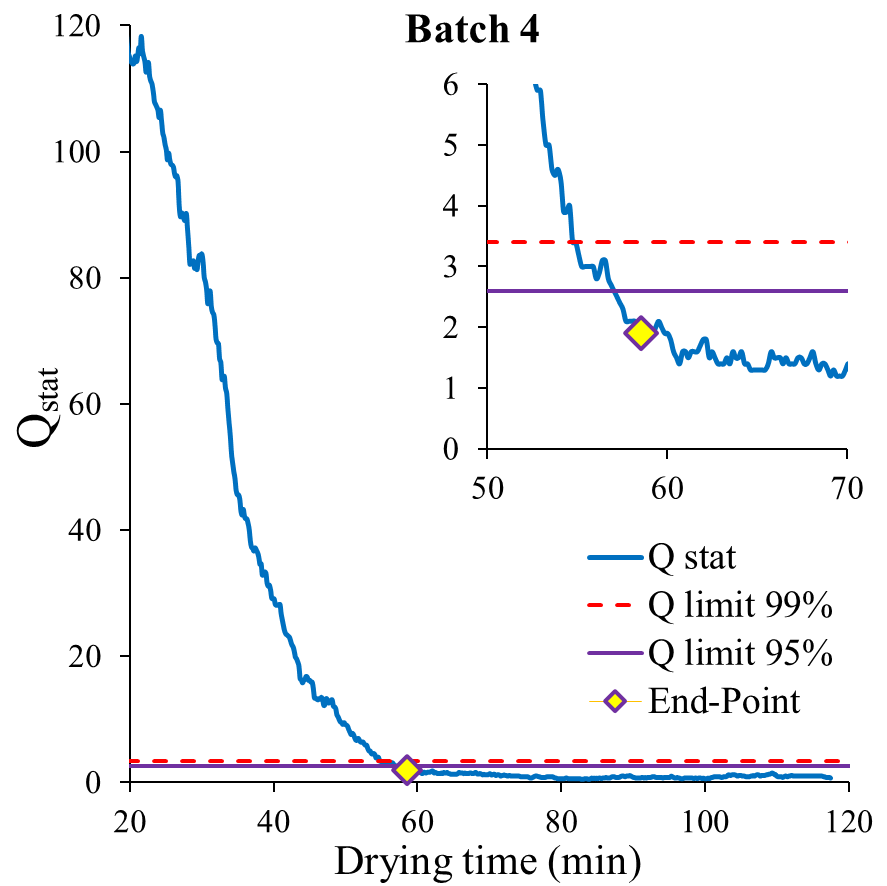


**Fig. S4.1.** MSPC control charts for batches 1, 2, 3 and 4. Inserted figures show in detail the final time range of the drying process and the process end-point, identified when 10 consecutive observations of Q_stat_ values were below the 95% control limit.


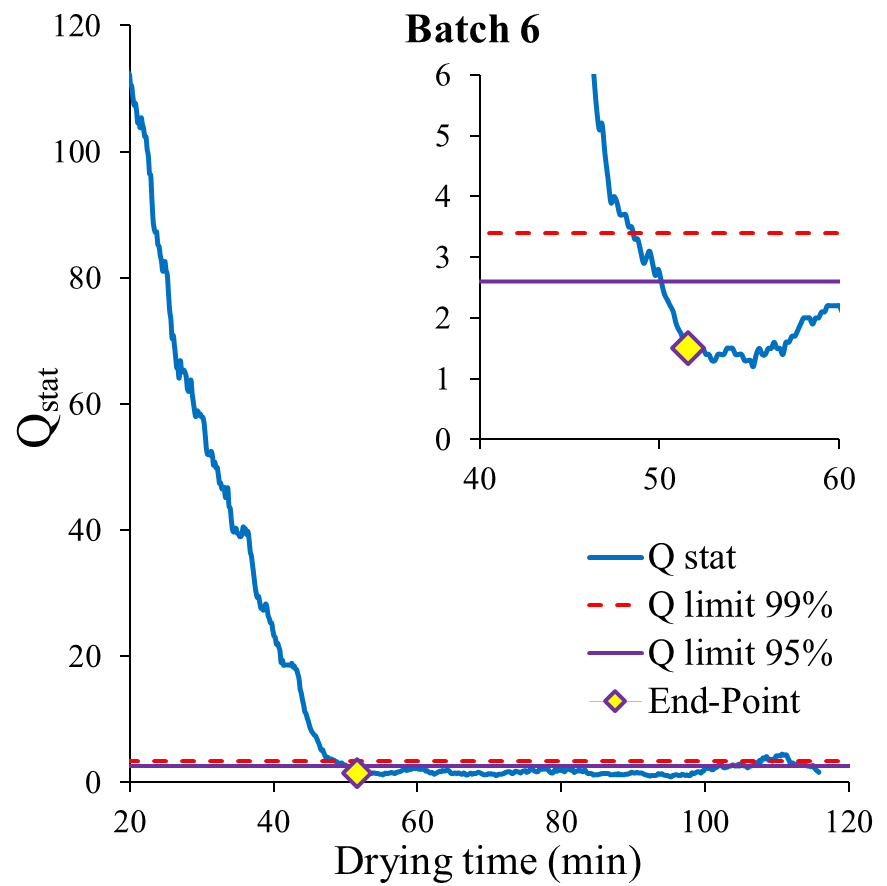

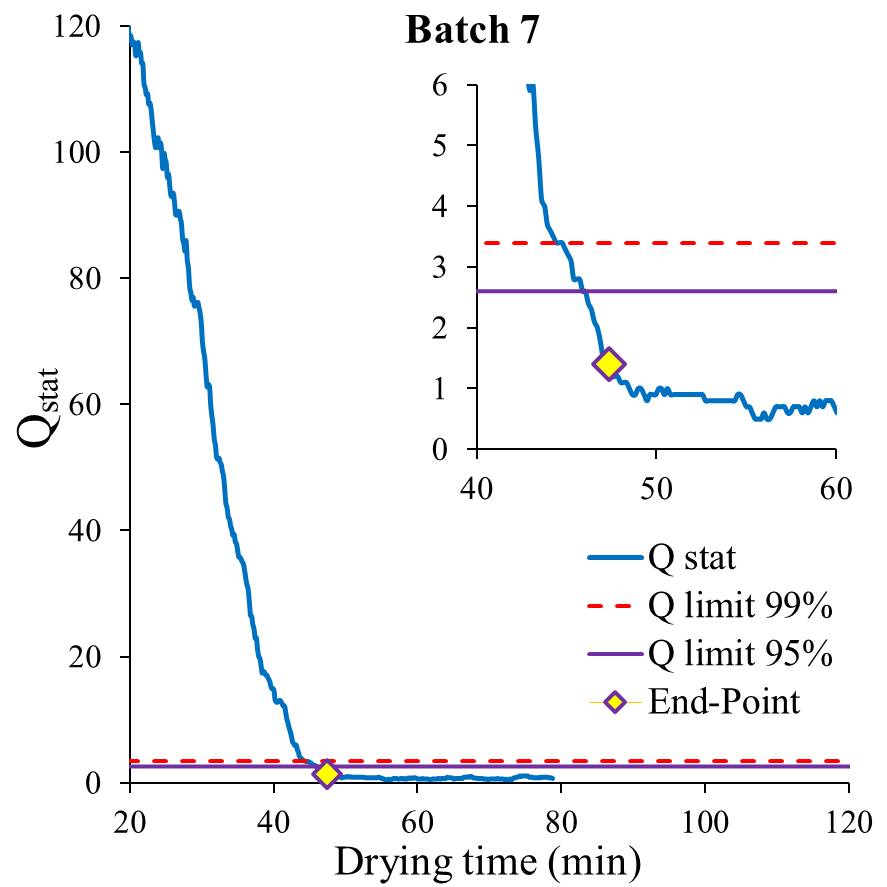

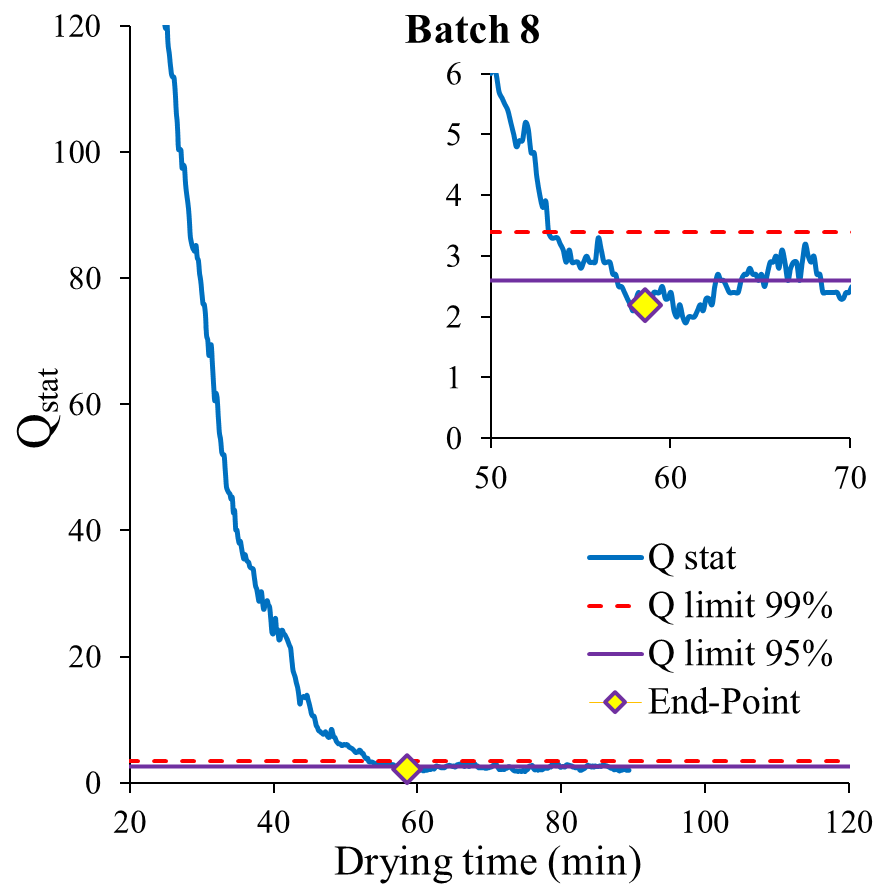

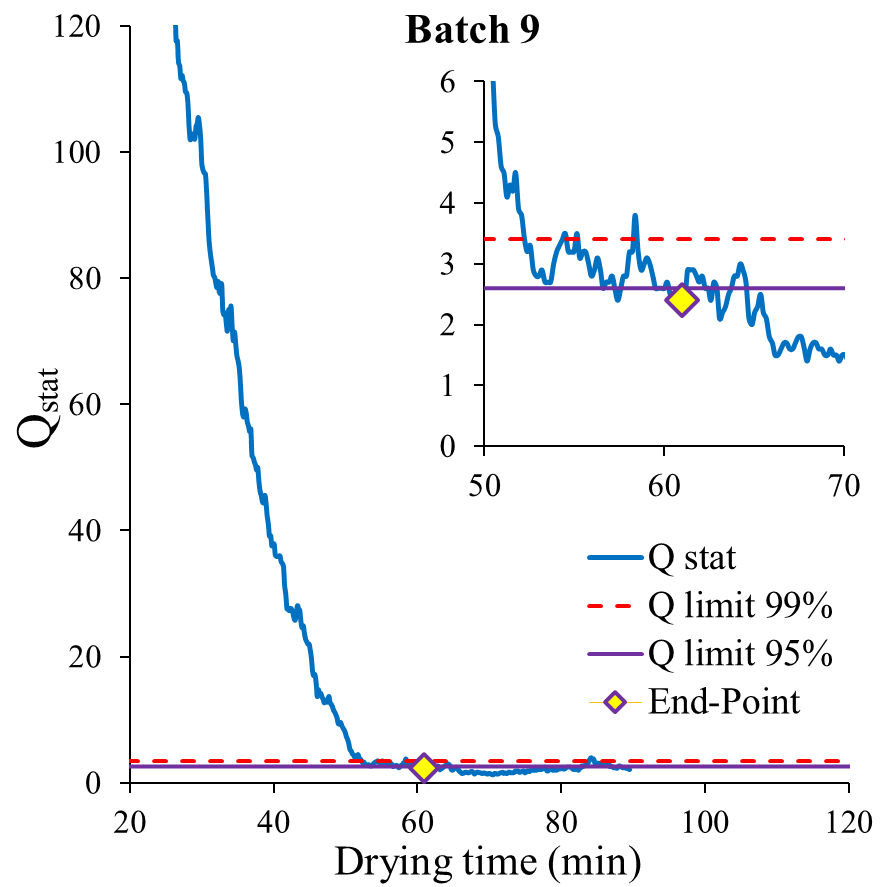


**Fig. S4.2.** MSPC control charts for batches 6, 7, 8, and 9. Inserted figures show in detail the final time range of the drying process and the process end-point, identified when 10 consecutive observations of Q_stat_ values were below the 95% control limit.


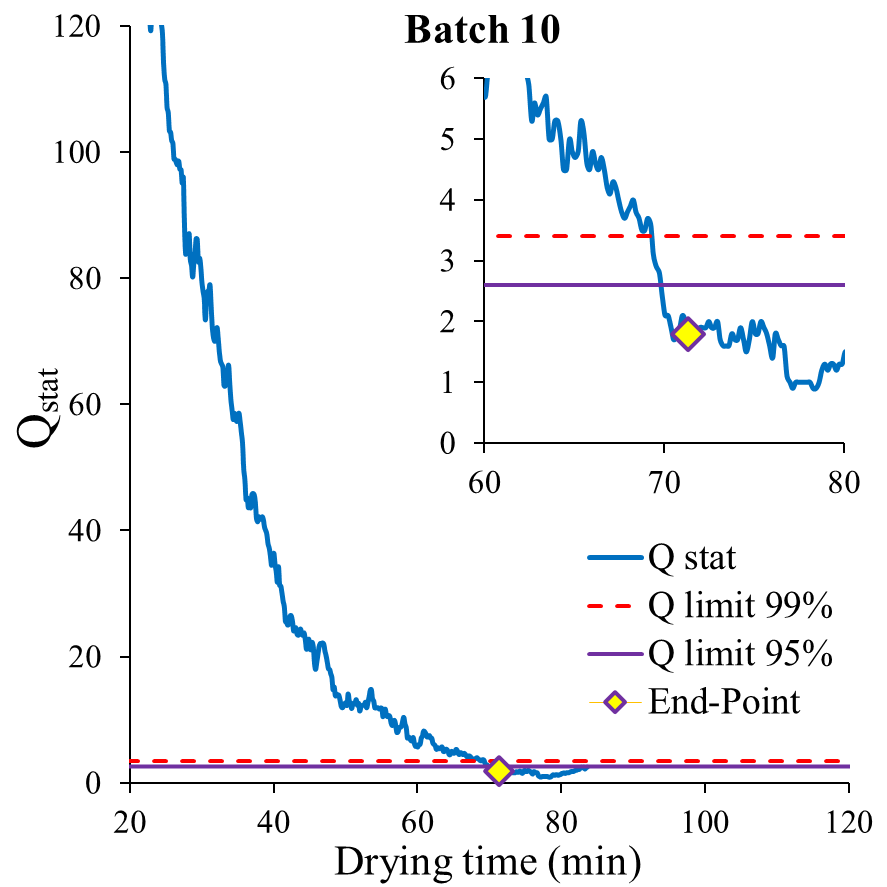

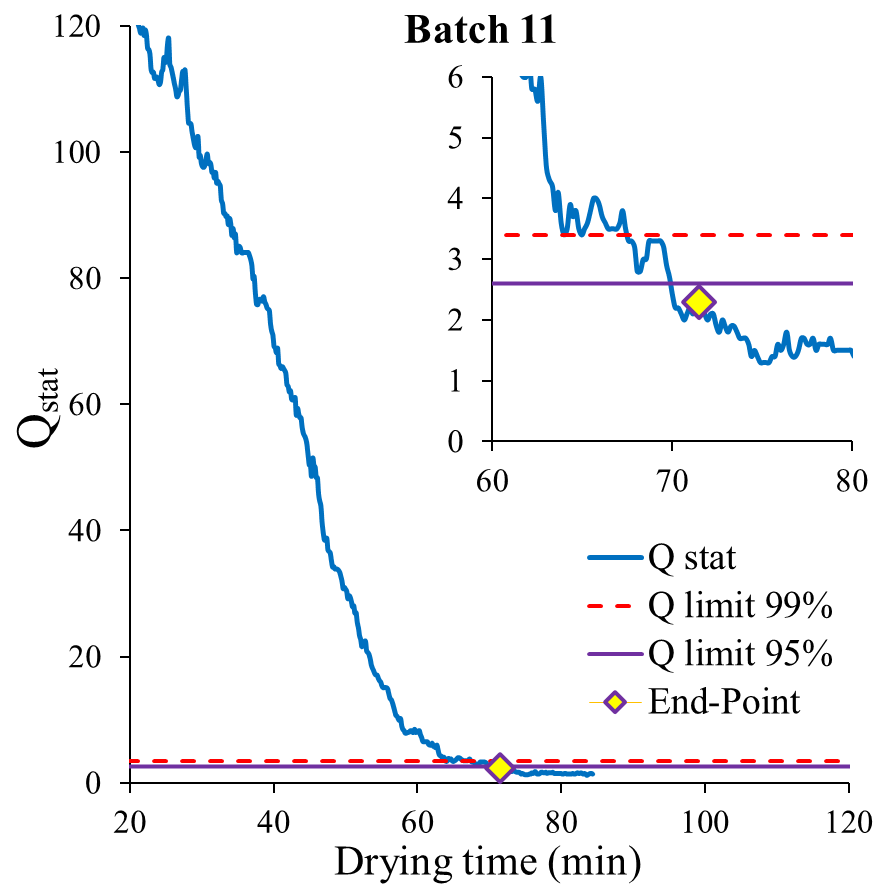

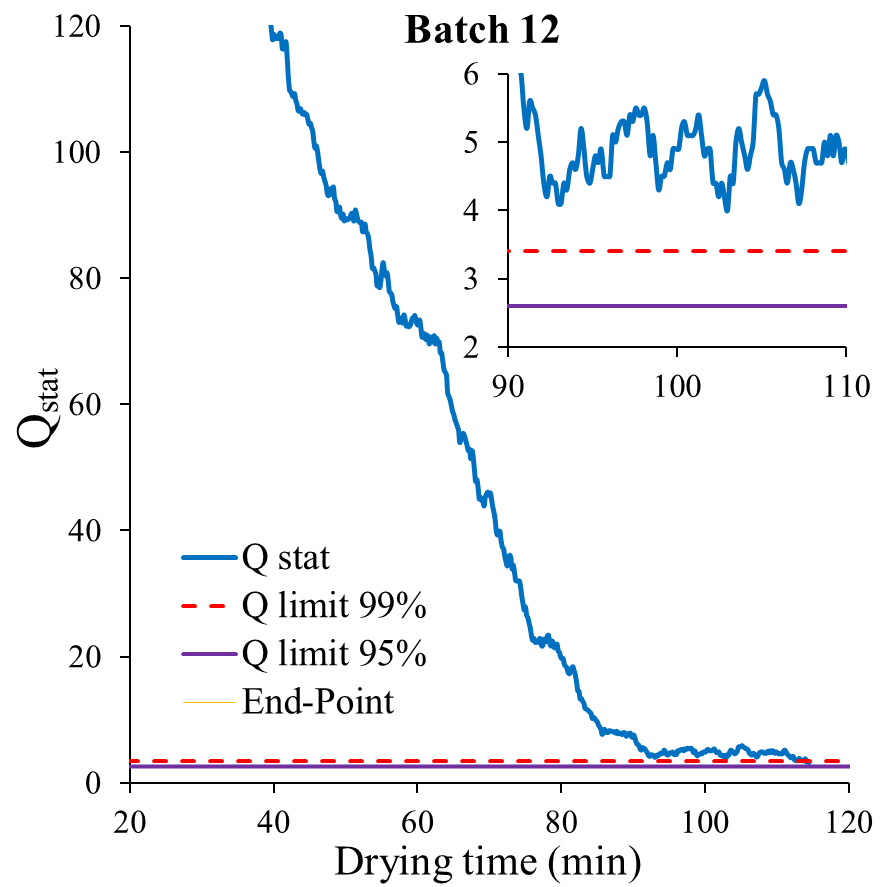

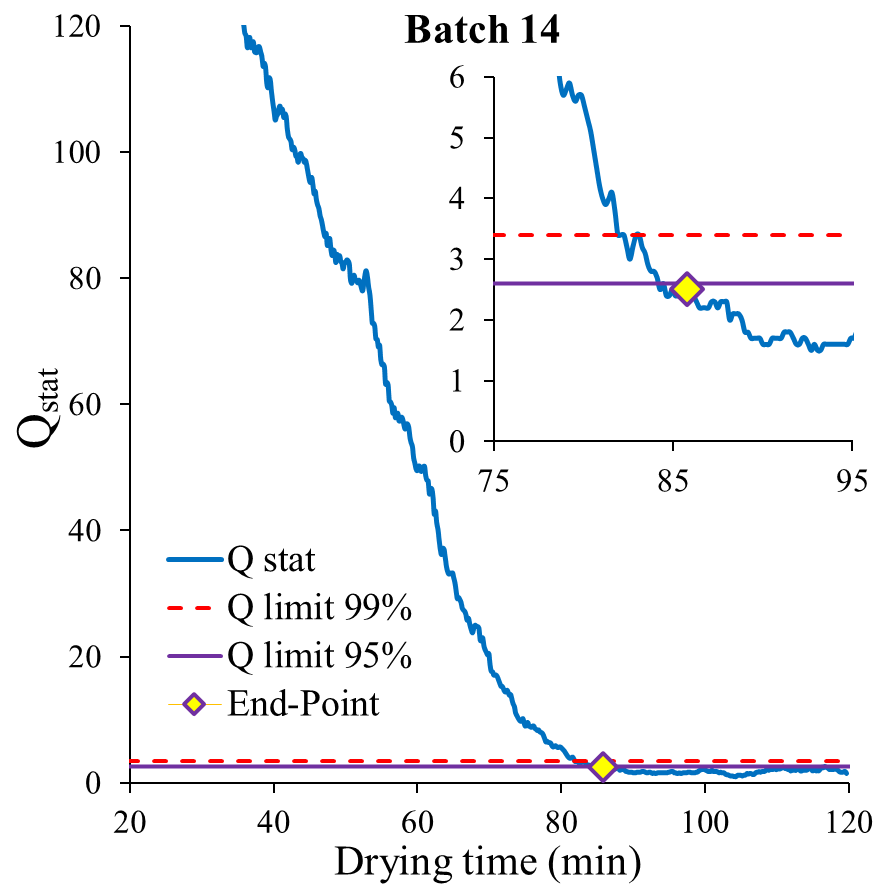


**Fig. S4.3.** MSPC control charts for batches 10, 11, 12, and 14. Inserted figures show in detail the final time range of the drying process and the process end-point, identified when 10 consecutive observations of Q_stat_ values were below the 95% control limit.


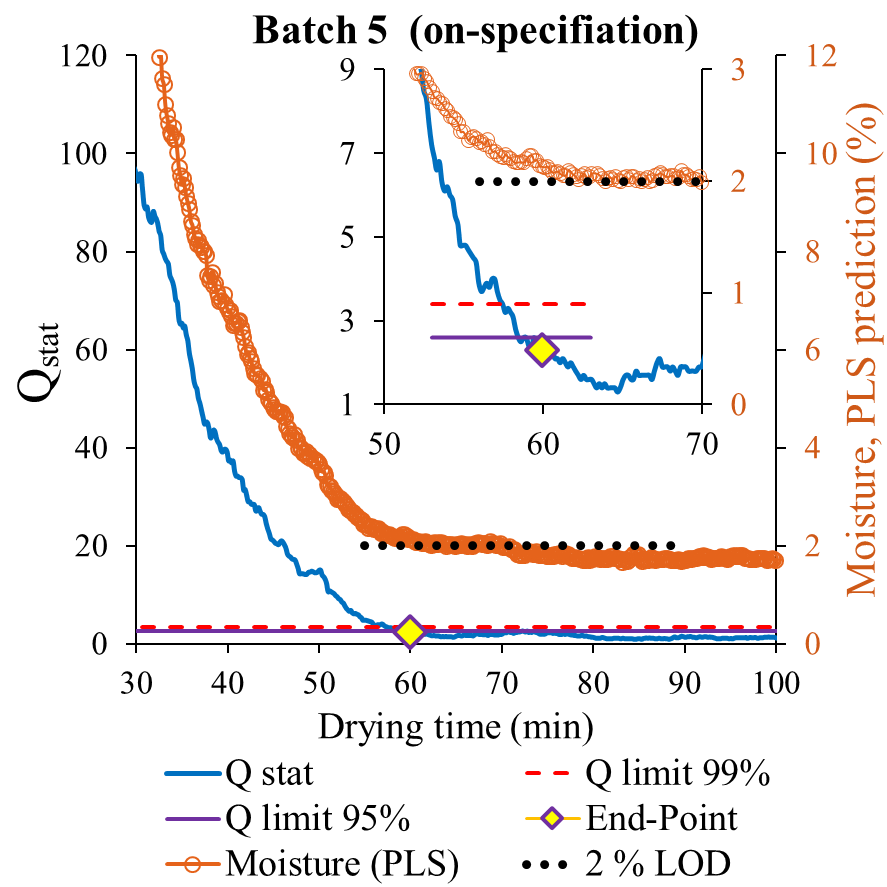

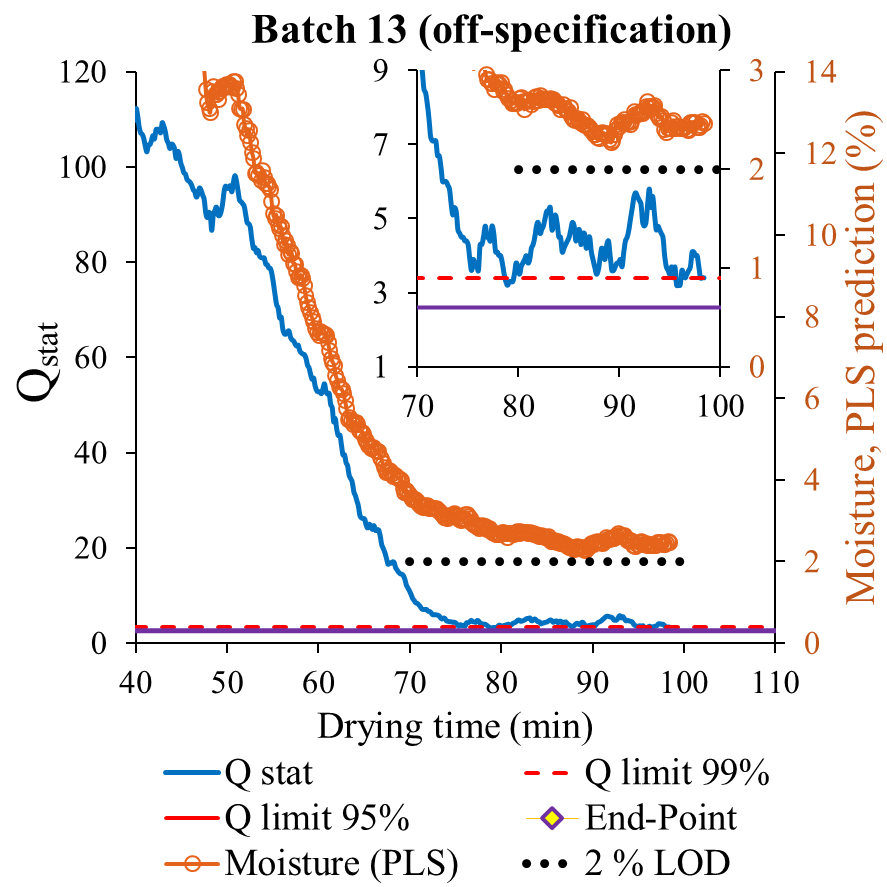


**Fig. S4.4.** MSPC control charts for batches 5 (on-specification) and 13 (off-specification), including the moisture predictions from PLS model for reference (secondary axis). Inserted figures show in detail the final time range of the drying process and the process end-point, identified when 10 consecutive observations of Q_stat_ values were below the 95% control limit.

**Fig. S4.5.** Loadings plot of the 2 principal components used in the PCA-based MSPC model for end-point detection.

For MSPC models the loading plot is given for completeness. Loading plots are very informative when PCA is done on the whole process trajectory, but this is not the case for MSPC. In the case of MSPC, the associated PCA model describes variations from the dry product only; virtually identical spectra that form the reference for the end-point detection.

PC1 marks the inverted water band and PC2, with a less interpretable shape, is required for the description of batch-to-batch variability.

# S5. Adsorbtion Isotherms

Isotherms relate the water activity ($a_{w}$) to water content, usually expressed on a dry weight bases $(F_{w}=m_{w}/m_{s})$. Isothems are typically fitted to an extended BET equation developed by Guggenheim, Andersen and de Boer and (de Boer 1968, Zografi 1988. The GAB correlation, expresses water content of solid i $\left( F_{w_{i}} \right)$as function of the water activity (table 1, figure X):

$$F_{w_{i}}\left( a_{w} \right)=\frac{m_{w_{i}}}{m_{s}}=\frac{{k_{w}}_{i}a_{w}C_{GAB_{i}}m_{o_{i}}}{\left( 1-k_{w_{i}}a_{w} \right)\left( 1+\left( C_{GAB_{i}}-1 \right)k_{w_{i}}a_{w} \right)}$$

Here ${k_{w}}_{i}, C_{GAB_{i}}, and m_{o_{i}}$ are material specific parameters. Note the inverse of the GAB equation follows from iteration of

$$a_{w}\left( F_{w_{i}} \right)_{n+1}=\left\{ \begin{matrix} a_{w_{n}}\leq0 & 0.5 a_{w_{n-1}} \\ otherwise & F_{w_{i}}\frac{\left( 1-k_{w_{i}}a_{w_{n}} \right)\left( 1+\left( C_{GAB_{i}}-1 \right)k_{w_{i}}a_{w_{n}} \right)}{{k_{w}}_{i}C_{GAB_{i}}m_{o_{i}}} \\ a_{w_{n}}\geq1 & 0.5a_{w_{n-1}}+0.5 \end{matrix} \right.$$

Typically 10 iterations are sufficient to converge to 0.0001.

If it is assumed that the three main solid-water phases within a single granule are in equilibrium with each other, we can define an aggregate isotherm to arrive at the water activity in granules with a certain moisture content. Assuming that mannitol does not alter the solid-water isotherms, and with $m_{i}$ the mass of adsorbent material i, and $F_{w_{i}}(a_{w})$ the isotherm of that material the aggregate isotherm is the mass based average of the isotherms:

$$F_{w}\left( a_{w} \right)=\frac{\sum_{i} m_{i} F_{w_{i}}\left( a_{w} \right)}{\sum_{i} m_{i}}$$

The aggregate isotherm can be fitted with the GAB correlation (Fig Xb, table y). and its inverse provides the water activity $a_{w}$ in the granules at certain %water.


Figure Y: Isotherm data and the corresponding GAB fit

de Boer, J. H. The dynamic character of adsorption, 2nd ed.; Clarendon Press: Oxford, 1968.

Zografi G. (1988) States of Water Associated with Solids,
Drug Development and Industrial Pharmacy, 14:14, 1905-1926, DOI: [10.3109/03639048809151997](https://doi.org/10.3109/03639048809151997)

Lin L, Quan G., Peng T., Huang Z., Singh V., Lu M., Wu C., (2017).
International Journal of Pharmaceutics, 533(1), 84-92.

Roja J., Moren S., Lopez A 2011. J. Pharm. Sci. & Res. Vol.3(7), 1302-1309

Faroongsarng D, Peck G.E. (1994) The Swelling & Water Uptake of Tablets III: Moisture Sorption Behavior of Tablet Disintegrants, Drug Development and Industrial Pharmacy, 20:5, 779-798, DOI: 10.3109/03639049409038331

Aspler J.S., Gray D.G. (1981) Mixing of water with(hydroxypropyl)cellulose liquid crystalline mesophases. Macromolecules 1981 14 (5), 1546-1549. DOI: 10.1021/ma50006a078

Table X: Isotherm data from references

# S6. Water vapour pressure in pharma dryers

The equilibrium vapour pressure of pure water at the temperatures typical for the drying of pharmaceutical products, 10 to 80 $℃$ has been fitted to within 1 Pa:

$P_{w}^{*}\left( T(℃) \right)=P_{W}^{*}(20℃) e^{-B*\left( \frac{1}{C+T}-\frac{1}{C+20} \right)}=2339.5*e^{-4078.8*\left( \frac{1}{236.63+T}-\frac{1}{256.63} \right)}\pm1 Pa$ (#1)

To ensure accuracy in the temperature range typical of dryers for pharmaceutical materials, the above equation is fitted to vapour pressure data from 0 to 50 $℃$ (Buck 1981) by minimising the sum of the relative and absolute sum of squares:

$$\min_{P_{W}^{*}\left( 20℃ \right), B, C} \sum_{i} \left( \frac{P_{w}^{*}\left( T_{i},P_{W}^{*}\left( 20℃ \right), B, C \right)}{P_{data}(T_{i})}-1 \right)^{2}+\sum_{i} \left( P_{w}^{*}\left( T_{i},P_{W}^{*}\left( 20℃ \right), B, C \right)-P_{data}\left( T_{i} \right) \right)^{2}$$

This objective function ensures that both the extremes are accurate.

The resulting fit is compared to other the buck 1981 equation (w6 in table 3 of Buck 1981), as well as two correlations presented by NIST. The tables below show that equation #1 is accuirate =/1 1 Pascal between 0 and 50 $℃$, and to within 1 % betyween -20 and 100 $℃$.

Figure X.Y.1: Details of the vapour pressure fit. The results of the fit from this work closely matches the correlation presented by Buck 1981. The correlations provided by NIST give quite large errors at lower temperatures, and significant errors (<5%) near the boiling point

**References**:

Buck, A.L., 1981: [New Equations for Computing Vapor Pressure and Enhancement Factor.](https://journals.ametsoc.org/doi/abs/10.1175/1520-0450%281981%29020%3C1527%3ANEFCVP%3E2.0.CO%3B2) *J. Appl. Meteor.,* **20**, 1527–1532, [https://doi.org/10.1175/1520-0450(1981)020<1527:NEFCVP>2.0.CO;2](https://doi.org/10.1175/1520-0450(1981)020%3c1527:NEFCVP%3e2.0.CO;2)

NIST <https://webbook.nist.gov/cgi/cbook.cgi?ID=C7732185&Mask=4>. correlation based on data from:

Daniel R. Stull (1947) Inorganic Compounds. Ind. Eng. Chem., 39 (4), 540–550.
DOI: 10.1021/ie50448a023

O. C. Bridgeman and E. W. Aldrich (1964). Vapor Pressure Tables for Water. J. Heat Transfer 86(2), 279-286.
doi:10.1115/1.3687121

| Table X.Y.1: Vapour pressure curves fits optimised for drying of Pharmaceutical products | | | | | | | | | |  |  |
| --- | --- | --- | --- | --- | --- | --- | --- | --- | --- | --- | --- |
|  | **Data** | **fit 1** | | **fit 2** | | **fit 3** | | **fit 4** | | | |
|  |  | *this work* | | *Buck 1981* | | *NIST Bridgeman &  Aldrich 1964* | | *NIST Stull 1947* | | | |
|  |  |  | |  | |  | |  | | | |
|  | $P_{w}^{*}$ | $P_{w}^{*}\left( 20℃ \right) e^{-B\left( \frac{1}{C+T(℃)}+\frac{1}{C+20} \right)}$ | | $P_{W}^{*}\left( 0℃ \right) e^{\left( B-\frac{T}{C1} \right)*\left( \frac{T}{T+C2} \right)}$ | | ${10}^{5} e^{A- \frac{B}{C+T(K)}}$ | | ${10}^{5} e^{A- \frac{B}{C+T(K)}}$ | | | |
|  |  |  |  |  |  |  |  |  |  | | |
|  |  | $P_{w}^{*}\left( 20℃ \right)$ | 2339.10 Pa | $P_{w}^{*}\left( 0℃ \right)$ | 611.210 Pa | A | 5.402210 | A | 4.654300 | | |
|  |  | B | 4078.80 | B | 18.5640 | B | 1838.6750 | B | 1435.2640 | | |
|  |  | C | 236.630 | C1 | 255.570 | C | -31.7370 | C | -64.8480 | | |
|  |  |  |  | C2 | 254.400 |  |  |  |  | | |
|  |  | min T | 0 C | min T | 0 C | min T | 273 K | min T | 273 K | | |
|  |  | max T | 50 C | max T | 100 C | max T | 303 K | max T | 373 K | | |
| T | P*w | P*w calc | delta | P*w calc | delta | P*w calc | delta | P*w calc | delta | | |
| C | Pa | Pa | Pa | Pa | Pa | Pa | Pa | Pa | Pa | | |
| 0 | 611.21 | 610.44 | -0.77 | 611.21 | 0.00 | 604.18 | -7.03 | 574.16 | -37.05 | | |
| 10 | 1227.90 | 1227.93 | 0.03 | 1227.81 | -0.09 | 1214.73 | -13.17 | 1188.80 | -39.10 | | |
| 20 | 2338.50 | 2339.10 | 0.60 | 2338.00 | -0.50 | 2315.10 | -23.40 | 2309.28 | -29.22 | | |
| 30 | 4245.20 | 4245.51 | 0.31 | 4243.98 | -1.22 | 4207.36 | -37.84 | 4242.56 | -2.64 | | |
| 40 | 7381.30 | 7380.64 | -0.66 | 7379.49 | -1.81 | 7328.26 | -53.04 | 7421.47 | 40.17 | | |
| 50 | 12345.00 | 12345.27 | 0.27 | 12343.47 | -1.53 | 12286.96 | -58.04 | 12431.88 | 86.88 | | |
|  |  |  |  |  |  |  |  |  |  | | |
| max error |  |  | 0.77 |  | 1.81 |  | 58.04 |  | 86.88 | | |
| stdev error |  |  | 0.56 |  | 0.77 |  | 21.01 |  | 50.72 | | |
|  |  |  |  |  |  |  |  |  |  | | |
|  |  |  |  |  |  |  |  |  |  | |  |

| Table x.y.2: Comparison of vapour pressure fits to correlation over 20-100C | | | | | | | | | | | | | | |  |
| --- | --- | --- | --- | --- | --- | --- | --- | --- | --- | --- | --- | --- | --- | --- | --- |
|  | fit 1 | | | fit 2 | | | fit 3 | | | | fit 4 | | | | |
|  | *this work* | | | *Buck 1981* | | | *NIST Bridgeman and Aldrich 1964* | | | | *NIST Stull 1947* | | | | |
| $P_{w}^{*}=$ | $P_{w}^{*}\left( 20℃ \right) e^{-B\left( \frac{1}{C+T(℃)}+\frac{1}{C+20} \right)}$ | | | $P_{W}^{*}\left( 0℃ \right) e^{\left( B-\frac{T}{D} \right)*\left( \frac{T}{T+C} \right)}$ | | | ${10}^{5} e^{A- \frac{B}{C+T(K)}}$ | | | | ${10}^{5} e^{A- \frac{B}{C+T(K)}}$ | | | | |
|  | $P_{w}^{*}\left( 20℃ \right)$ | | 2339.10 Pa | $P_{w}^{*}\left( 0℃ \right)$ | 611.210 Pa | | A | 5.402210 | | | A | | 4.654300 | | |
|  | B | | 4078.80 | B | 18.5640 | | B | 1838.6750 | | | B | 1435.2640 | | | |
|  | C | | 236.630 | C | 255.570 | | C | -31.7370 | | | C | -64.8480 | | | |
|  |  | |  | D | 254.400 | |  |  | | |  | |  | | |
|  | min T | | 0 C | min T | 0 C | | min T | 273 K | | | min T | | 273 K | | |
|  | max T | | 50 C | max T | 100 C | | max T | 303 K | | | max T | | 373 K | | |
|  |  | |  |  | | |  | |  | |  | |  | | |
| T | P*w calc | delta | | *P*w calc (reference)* | | | P*w calc | | | delta | P2w calc | | | delta | |
| C | Pa | Pa | | *Pa* | |  | Pa | | | Pa | Pa | | | Pa | |
| -30 | 50.0 | -1.98% | | *51.0* | |  | 50.0 | | | -1.94% | 39.6 | | | -22.30% | |
| -20 | 124.3 | -1.01% | | *125.6* | |  | 123.7 | | | 16.47% | 106.2 | | | -15.44% | |
| -10 | 285.3 | -0.43% | | *286.6* | |  | 282.9 | | | 9.79% | 257.7 | | | -10.08% | |
| 0 | 610.4 | -0.13% | | *611.2* | |  | 604.2 | | | 5.23% | 574.2 | | | -6.06% | |
| 10 | 1228 | 0.01% | | *1228* | |  | 1215 | | | 2.18% | 1189 | | | -3.18% | |
| 20 | 2339 | 0.03% | | *2338* | |  | 2315 | | | 0.25% | 2309 | | | -1.24% | |
| 30 | 4246 | 0.01% | | *4245* | |  | 4207 | | | -0.83% | 4243 | | | -0.06% | |
| 40 | 7381 | -0.02% | | *7382* | |  | 7328 | | | -1.26% | 7421 | | | 0.53% | |
| 50 | 12345 | -0.03% | | *12349* | |  | 12287 | | | -1.17% | 12432 | | | 0.67% | |
| 60 | 19945 | 0.00% | | *19945* | |  | 19906 | | | -0.66% | 20039 | | | 0.47% | |
| 70 | 31232 | 0.10% | | *31201* | |  | 31266 | | | 0.18% | 31211 | | | 0.03% | |
| 80 | 47539 | 0.27% | | *47410* | |  | 47746 | | | 1.29% | 47138 | | | -0.57% | |
| 90 | 70523 | 0.53% | | *70152* | |  | 71074 | | | 2.63% | 69252 | | | -1.28% | |
| 100 | 102197 | 0.88% | | *101308* | |  | 103361 | | | 4.16% | 99232 | | | -2.05% | |
| 110 | 144959 | 1.32% | | 143068 | |  | 147144 | | | 5.85% | 139010 | | | -2.84% | |
|  |  |  | |  | |  |  | | |  |  | | |  | |

**Figure S7.1:** **(A)** a granule consists of solid held together by liquid bridges formed by the binder fluid. Water is also absorbed by solids (~0.3 gr water/gr solid). **(B)** The process scheme shows the location of water in different environments (“phases”) with arrows representing mass transfer between the environments. The gas phase concentration over (in equilibrium with) the solid ($C_{S}^{*}$) and liquid ($C_{L}^{*}$) are assumed to be similar. **(C)** The mass transfer may be represented as a resistance model with a “current” of water ($MTR_{H_{2}O},\frac{mol}{s.m_{bed}^{3}}$) flowing from high to low concentration. The transport through each environment requires a fall in concentration that is proportional to the “current”: $\Delta C=MTR_{H_{2}O}\times\Omega$, where $\Omega$ is the so called mass transfer resistance. The driving force (the “voltage”, the sum of all $\Delta C$) equals the concentration difference between the source of the water and the final sink, the fluidising gas: $C_{S}^{*}-C_{g}$.

# S7. Derivation of Fluid bed Resistance model

The mass transfer process can be represented with a resistance model ([37], figure S7.1C) that sees a “current” of water ($MTR_{w},\frac{mol}{s.m_{bed}^{3}}$) flowing from the high concentration at the source (liquid bridges, solids) to low concentration in the gas used to fluidised the bed. The transport through each environment requires a fall in concentration that is proportional to the “current”,
$\Delta C_{i}=MTR_{w}\times\Omega_{i}$, where $\Omega_{i}$ is the a called a *mass transfer resistance* which has the units of seconds. The overall driving force (equivalent to “voltage”) is the sum of all these $\Delta C_{i}$ and equals the concentration difference between the source of the water and its final sink, the fluidising gas:

| $C_{S}^{*}-C_{g}=\sum\Delta C_{i}=MTR_{H_{2}O}\times\sum\Omega_{i}$ | (7) |
| --- | --- |

The above equation shows the total mass transfer resistance $\Omega_{tot}=\sum\Omega_{i}$ to be the sum of the individual resistances, in similarity with Ohm’s law. As the residence time of the gas is short (< 100 ms) it is common in most fluidised bed models to assume that the mass transfer resistance $\Omega_{tot}$ and the bed’s temperature and moisture content are constant on the time scale required for the gas to flow from the bottom to the top of the bed. A mass balance over a horizontal slice of the bed with volume$dV_{bed}$:

| $\varphi_{g} dC_{g}=MRT_{w} dV_{bed}= MRT_{w}\frac{1}{f_{s}\rho_{s}}dm_{s}$ | (8) |
| --- | --- |

Here $f_{s}$is the volume fraction solids in the fluidised bed (estimated at 40%), and the $\rho_{s}$solids skeletal density (averaged at 1500 kg/m^3^). Substitution of eq 7 in 8 and integration yields (see ESI):

| $\varphi_{g} dC_{g}=\frac{1}{\Omega_{tot}}\left( C_{S}^{*}-C_{g} \right) \frac{1}{f_{s}\rho_{s}}dm_{s}$  $\int_{in}^{out} \frac{dC_{g}}{C_{S}^{*}-C_{g}}=\int_{0}^{m_{s}} \frac{1}{\Omega_{tot}} \frac{1}{{\varphi_{g}f}_{s}\rho_{s}}dm_{s}$  $-ln\left( \frac{C_{S}^{*}-C_{g.out}}{C_{S}^{*}-C_{g.in}} \right)=\frac{1}{\Omega_{tot}} \frac{1}{{\varphi_{g}f}_{s}\rho_{s}}m_{s}$ |  |
| --- | --- |

| $\begin{matrix} C_{g.out}-C_{g.in}= & \left( 1-S_{in} \right) & \left( 1-e^{- \frac{m_{s}}{\Omega_{tot}f_{s}\rho_{s}\varphi_{g}}} \right) C_{S}^{*} \\ & & f_{MTR} \end{matrix}$ | (9) |
| --- | --- |

Here $S_{in}=C_{g.in}/C_{S}^{*}$ is the degree of saturation of the inlet gas which varies between 0 (no water) to 1 for an inlet gas in equilibrium with the water in the granules. It is important to realise that the saturation of the inlet gas ($S_{in}$) may change during processing, as $C_{S}^{*}$ varies with both bed temperature and water content. We estimated $C_{g.in}$ such that the outlet air is saturated at the beginning of the constant drying rate period.

The molar drying rate $\dot{N}_{w}$ in the fluid bed dryer follows from the mass balance over the unit:

| $\dot{N}_{w}=\left( C_{g.out}-C_{g.in} \right)\times\varphi_{g}$ |  |
| --- | --- |

Substituting eq (9)

| $\dot{N}_{w}=\left( 1-S_{in} \right)\left( 1-e^{- \frac{m_{s}}{\Omega_{tot}f_{s}\rho_{s}\varphi_{g}}} \right) C_{S}^{*}\times\varphi_{g}$ |  |
| --- | --- |

With:

$$f_{MTR}=1-e^{- \frac{m_{s}}{\Omega_{tot}f_{s}\rho_{s}\varphi_{g}}}$$

It follows that:

| $\dot{N}_{w}=\left( C_{g.out}-C_{g.in} \right)\times\varphi_{g}= f_{MTR}\times\left( 1-S_{in} \right) C_{S}^{*} \varphi_{g}$ | (10) |
| --- | --- |

$f_{MTR}$ is the extent to which mass transfer is limiting: when $f_{MTR}=0$ the mass transfer resistances are high and no significant transfer occurs. If on the other hand $f_{MTR}=1$ then mass transfer is instantaneous, and the gas phase leaves saturated resulting in the maximum drying rate $\dot{N}_{w}^{\infty}$. The 2^rd^ row in figure 11 shows the molar and maximum drying rates. The drying rate is about 80% of the maximum drying rate in the constant rate period which ends at $F_{w}\approx0.35$, after which the rate reduces in a manner that appears proportional with $F_{w}$. Conversely, the maximum drying rate remains stable at $F_{w}<0.35$, as $T_{bed}$ and $P_{W}^{*}(T_{bed})$ increase balanced by a reduction in $a_{w}$ as water is removed. The reduction of $a_{w}$ becomes dominate when $F_{w}<0.1$ the driving force and drying rates reduce then sharply.

Step 4 calculate the overall mass transfer resistance

$\dot{N}_{w}^{\infty}$ is the maximum drying rate, that would be achieved when resistance goes to zero:

$$\lim_{\Omega_{tot}\to0} 1-e^{- \frac{m_{s}}{\Omega_{tot}f_{s}\rho_{s}\varphi_{g}}}=1-0=1$$

$$\dot{N}_{w}^{\infty}=\lim_{\Omega_{tot}\to0} \dot{N}_{w}=\left( 1-S_{in} \right) C_{S}^{*}\times\varphi_{g}$$

In other words, $\dot{N}_{w}^{\infty}$ is the drying rate acheieved when the gas flowing through the dryer reaches equilibrium with the material in the dryer. The mass transfer resistance $\Omega_{tot}$ follows from the ratio of the molar drying rate $\dot{N}_{w}$ measured by NIR, and the maximum drying rate $\dot{N}_{w}^{\infty}.$

| $f_{MTR}=\frac{\dot{N}_{w}}{\dot{N}_{w}^{\infty}}=\frac{\left( 1-S_{in} \right)\left( 1-e^{- \frac{m_{s}}{\Omega_{tot}f_{s}\rho_{s}\varphi_{g}}} \right) C_{S}^{*}\times\varphi_{g}}{\left( 1-S_{in} \right) C_{S}^{*} \varphi_{g}}=1-e^{- \frac{m_{s}}{\Omega_{tot}f_{s}\rho_{s}\varphi_{g}}}$ | (11) |
| --- | --- |

The measured temperature and water content data combined with the aggregate isotherm and the vapour pressure of water allows calculation of $f_{MTR}$. The overall mass transfer resistance then follows by rearranging

$$f_{MTR}=1-e^{- \frac{m_{s}}{\Omega_{tot}f_{s}\rho_{s}\varphi_{g}}}$$

$$\ln{(1-f}_{MTR})=- \frac{m_{s}}{\Omega_{tot}f_{s}\rho_{s}\varphi_{g}}$$

| $\Omega_{tot}=-\frac{m_{s}}{Ln\left( 1-f_{MTR} \right) f_{s}\rho_{s}\varphi_{g}}$ | (12) |
| --- | --- |

# Acknowledgments

The presented work has received funding from the ProPAT Project (European Union Horizon 2020 research and innovation programme under grant agreement 637232). The authors also thank Spectral Engines (Finland) for facilitating the MEMS-FPI sensors used in this study, and particularly Uula Kantojärvi and Matti Tammy for their technical support.
